# yEvo: A modular eukaryotic genetics and evolution research experience for high school students

**DOI:** 10.1002/ece3.10811

**Published:** 2024-01-07

**Authors:** M. Bryce Taylor, Alexa R. Warwick, Ryan Skophammer, Josephine M. Boyer, Renee C. Geck, Kristin Gunkelman, Margaux Walson, Paul A. Rowley, Maitreya J. Dunham

**Affiliations:** ^1^ Department of Genome Sciences University of Washington Seattle Washington USA; ^2^ Program in Biology Loras College Dubuque Iowa USA; ^3^ Department of Fisheries and Wildlife Michigan State University East Lansing Michigan USA; ^4^ Westridge School Pasadena California USA; ^5^ Department of Biological Sciences University of Idaho Moscow Idaho USA; ^6^ Department of Teacher Education Michigan State University East Lansing Michigan USA

**Keywords:** evolution, genomics, research experience, secondary education, yeast

## Abstract

The resources for carrying out and analyzing microbial evolution experiments have become more accessible, making it possible to expand these studies beyond the research laboratory and into the classroom. We developed five connected, standards‐aligned yeast evolution laboratory modules, called “yEvo,” for high school students. The modules enable students to take agency in answering open‐ended research questions. In Module 1, students evolve baker's yeast to tolerate an antifungal drug, and in subsequent modules, investigate how evolved yeasts adapted to this stressful condition at both the phenotype and genotype levels. We used pre‐ and post‐surveys from 72 students at two different schools and post‐interviews with students and teachers to assess our program goals and guide module improvement over 3 years. We measured changes in student conceptions, confidence in scientific practices, and interest in STEM careers. Students who participated in yEvo showed improvements in understanding of activity‐specific concepts and reported increased confidence in designing a valid biology experiment. Student experimental data replicated literature findings and has led to new insights into antifungal resistance. The modules and provided materials, alongside “proof of concept” evaluation metrics, will serve as a model for other university researchers and K − 16 classrooms interested in engaging in open‐ended research questions using yeast as a model system.

## Graphical abstract

We present five yeast evolution and genomics lab modules for high school biology students to take agency in answering open‐ended research questions. Evaluations suggest improved student conceptions of evolution and genetics and increased confidence in some science practices.

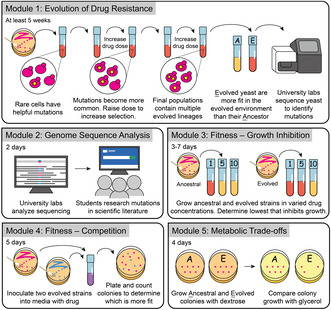

## 1 INTRODUCTION

A growing movement in STEM education aims to incorporate research experiences into the K‐16 classroom (Sadler & McKinney, 2010). These efforts have taken on many names, including student–scientist or school–scientist partnerships (SSPs; Clendening, 2004) and course‐based undergraduate research experiences (CUREs; Auchincloss et al., 2014; Krim et al., 2019). Research experiences seem to have positive impacts on many stakeholders (Laursen et al., 2007). For instance, participation in research can lead to improvements in students' confidence, grasp of concepts, and interest in STEM careers (Auchincloss et al., 2014; Hunt et al., 2021; Indorf et al., 2019; Krim et al., 2019). They may help with recruiting and retaining women and students from underrepresented backgrounds (Bangera & Brownell, 2014; Hunt et al., 2021) by allowing students to gain confidence in their ability to succeed in STEM settings (Hunt et al., 2021). Additionally, participation in research by teachers can have positive impacts on student achievement (Silverstein et al., 2009).

The value of classroom research experiences has led to a growing body of research and implementations at colleges and universities (see CUREnet https://serc.carleton.edu/curenet/index.html). Compared to the college level, there have been few published examples of research experiences in high school classrooms (Tanner et al., 2003; Ufnar & Shepherd, 2020). This paucity is in spite of the fact that at the K‐12 level, these efforts are in alignment with the Next Generation Science Standards (NGSS) (NGSS, 2013), which have made participation in the scientific process through inquiry‐based activities a central organizing principle in curriculum design.

Microbial experimental evolution, sometimes referred to as adaptive laboratory evolution, is appealing for classroom‐based research activities because these experiments have relatively low resource requirements and can be carried out in a few weeks with periodic short (10–20 min) interventions. Experimental evolution has been successfully used in teaching modules for K‐12 and college courses (Bennett et al., 2021; Cooper et al., 2019; Dahan et al., 2019; Ratcliff et al., 2014; Smith et al., 2016) and can connect concepts in evolution, cell biology, genetics, medicine, and biotechnology. These connections are relevant to the NGSS emphasis on “cross‐cutting concepts” that demonstrate commonalities between disciplines that are often taught separately.

In the experimental evolution framework, populations of organisms, most commonly microbes, are propagated under suboptimal conditions that restrict growth, such as elevated temperatures, exposure to drugs, or nutrient limitation. This imposes a strong selective pressure on the population. As a population of microbes is propagated in a suboptimal growth environment, rare spontaneous mutations that lead to enhanced growth rise in frequency due to natural selection until they constitute an increased fraction of the microbial population (reviewed in Kawecki et al., 2012; Lang & Desai, 2014; McDonald, 2019). This change results in phenotypic differences at the population level (i.e., increased growth), allowing experimenters to observe evolution in real time. Such experiments can demonstrate the reproducibility of a random evolution process toward a particular outcome. Though this is not the only mechanism of evolution, in our experience it is a powerful demonstration of evolution in action.

Whole‐genome sequencing of mutant organisms isolated from these experiments can provide insight into the genetic and molecular changes that lead to adaptation to a specific selective pressure (Bruger & Marx, 2018; Long et al., 2015; Payen et al., 2016). Traditionally this experimental paradigm has addressed basic evolutionary questions, such as the speed, dynamics, and limits of adaptation (e.g., Kawecki et al., 2012; Lenski, 2017; McDonald, 2019). It is increasingly implemented in applied contexts as a form of domestication to isolate microbes adapted to industrial settings (Giannakou et al., 2020; Lee & Kim, 2020; Sandberg et al., 2019).

For several reasons, the budding yeast *Saccharomyces cerevisiae* is a popular species for experimental evolution and an attractive microbial model system for high school students (Duina et al., 2014). (1) *S. cerevisiae* can be grown safely and easily within a classroom environment without specialized equipment. (2) It is one of the most well‐characterized organisms from a molecular and genetic standpoint due to extensive work both in laboratories and in broadly familiar settings such as baking and fermentation. (3) Its small, highly annotated genome, ease of phenotyping, and short generation time make it ideal for experimental evolution studies. (4) It is a key model organism that has yielded many discoveries about fundamental biological principles. It is also an important industrial organism used to produce proteins and small molecules for pharmaceutical and biotechnology applications. (5) Thousands of academic and industrial researchers are working with *S. cerevisiae* in both basic and applied research (e.g., Botstein & Fink, 2011; Lee & Kim, 2020).

**FIGURE 1:**
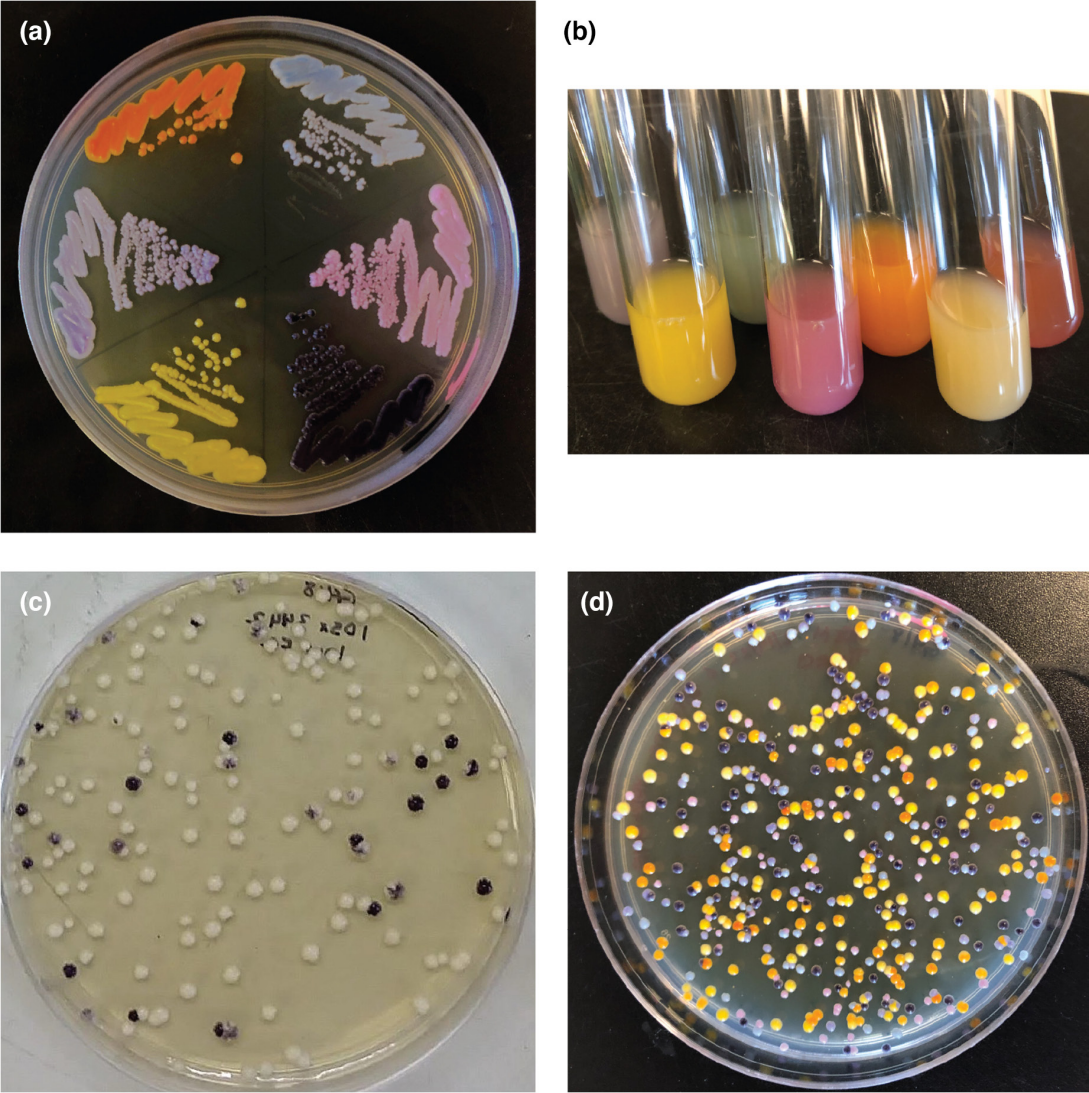
Yeast expressing different color pigments grown (a) on solid media and (b) in liquid culture. (c) Two or (d) six yeast strains expressing different pigments can be differentiated after co‐culture.

To address the need for authentic research experiences in high school that can be connected to learning objectives in evolution, cell biology, and genetics, we developed protocols and teaching materials for the experimental evolution of *S. cerevisiae* in a high school classroom setting, named “yEvo” (yeast Evolution). These experiments utilize strains of yeast engineered to express vibrant pigments (Figure 1). These strains provide many technical advantages for experimental use (Text S1), including a simple means to monitor for culture contamination or mislabeling, which are consistent issues in microbiology experiments. Given that adoption of scientist–teacher partnerships is often limited by suitability of time, our protocols were developed in close collaboration with high school teachers to ensure that they would be compatible with classroom learning objectives, teacher interests, and practical constraints (Saat et al., 2022). We leveraged the flexibility of our experimental system to develop multiple versions of our evolution protocol to suit a variety of time and resource availabilities. Our protocols differ from previous evolution teaching modules in that our experimental question is open‐ended, and the classroom projects have the opportunity to contribute to an ongoing research program. Key features in our design include opportunities for student agency (choosing aspects of experimental conditions for selection), competition (experiments to compare the fitness of evolved yeast), and collaboration (working together in teams, performing a literature search based on student mutation data).

Our yEvo lab experiments tasked students with identifying molecular factors that allow *S. cerevisiae* to gain resistance to an over‐the‐counter azole‐class antifungal drug called clotrimazole (Allen et al., 2015; Shafiei et al., 2020). This topic is highly relevant because antifungal resistance among pathogenic fungi is a growing global threat to human health and to food security (e.g. Berkow & Lockhart, 2017; Fisher et al., 2018). Research into the genetic basis of resistance to azole drugs has allowed medical researchers to develop new approaches to treat drug‐resistant pathogens (e.g., Cowen et al., 2009) and to predict which treatments are most likely to work for each patient's infection (e.g., Berkow & Lockhart, 2017; Cowen et al., 2015). *S. cerevisiae* is a safer alternative to experiments with pathogenic fungi as it cannot cause disease in healthy people yet shares many of the genes involved in antifungal resistance with pathogenic fungi.

**FIGURE 2:**
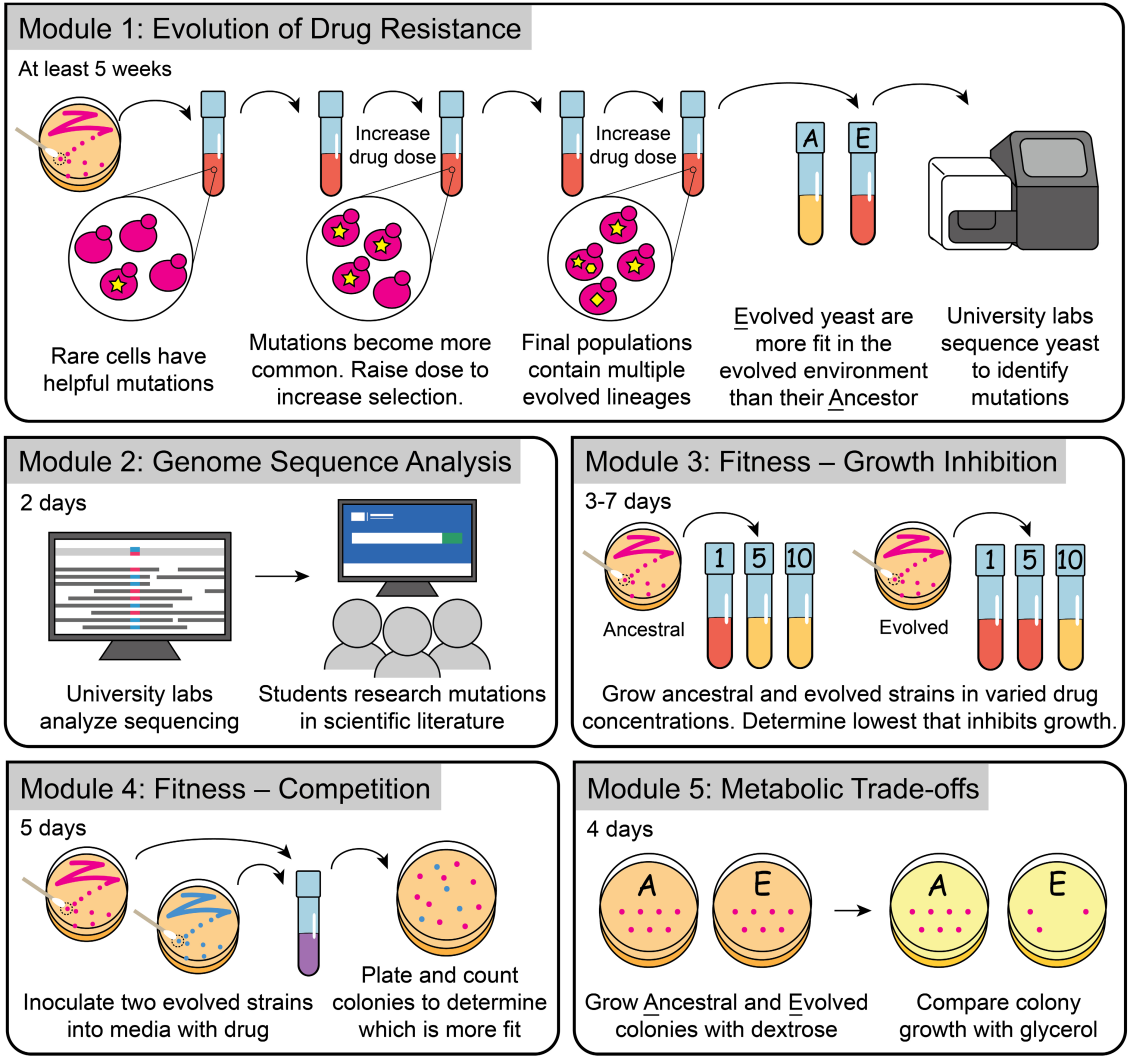
Overview of yEvo Modules 1–5.

This paper presents our yEvo teaching resources, organized into five laboratory modules (Figure 2) that can connect to various topics in standard high school biology curricula (e.g., College Board and NGSS; Table 1). We discuss reflections on our module design and improvement process over the first 3 years of implementation, which were guided by “proof of concept” evaluation metrics using student surveys and interviews with students and teachers. Survey questions measured changes in students' disciplinary knowledge, STEM career interest, confidence with scientific practices, and general engagement with the material in three high school teachers' classrooms in two different schools. Our partner teachers devoted class time for 15–34 weeks on Module 1, which could be daunting to new yEvo teachers. Thus, we also addressed two potential teacher concerns: (1) whether the amount of time required for these exercises detract from other educational goals, and (2) whether the repetitiveness of Module 1 impacted technical confidence and interest in biology/STEM careers. Student feedback across multiple years and schools was vital to our protocol and curriculum revision. We expect this collaborative endeavor will serve as a model for other university researchers and high school teachers interested in engaging K‐12 students in authentic research experiences.

**TABLE 1:** Curricular overview of yEvo modules with connections to common United States science standards.

Module	Lab skills	Suggested discussion topics	Example learning objective	Next Generation Science Standards	College Board learning objectives
1: Evolution of Drug Resistance	Sterile technique, record‐keeping, culturing microbes	Evolution, selection, mutation, mechanisms of drug action (azoles)	Apply microbial culture practices that select for altered phenotypes	HS‐LS4‐1 HS‐LS4‐2 HS‐LS4‐4	LO 1.2 LO 1.26
2: Genome Sequence Analysis	Data analysis, literature search, mechanisms of drug action (azoles)	Genetics, genomics, mutation, gene regulation, metabolism, cell membrane structure, use of model organisms	Formulate connections between DNA sequence changes and organism‐level phenotypes	HS‐LS1‐1 HS‐LS3‐1 HS‐LS3‐2 HS‐LS4‐1 HS‐LS4‐4	LO 3.20 LO 3.24 LO 3.25 LO 3.6
3: Fitness ‐ Growth Inhibition	Sterile technique, serial dilutions, data analysis, controlled experiment, statistics, culturing microbes	Selection, fitness	Measure differences in phenotype between multiple strains of microbe	HS‐LS4‐2	LO 1.2 LO 1.26
4: Fitness – Competition	Sterile technique, serial dilutions, data analysis, controlled experiment, statistics, culturing microbes	Selection, fitness	Measure differences in fitness between multiple strains of microbe	HS‐LS4‐2	LO 1.2 LO 1.26
5: Metabolic Trade‐offs	Sterile technique, statistics, isolating individual microbes	Metabolism, trade‐offs, mitochondrial function	Understand how an organism's fitness is specific to one environment	HS‐LS1‐7 HS‐LS4‐5	LO 1.2 LO 1.26

*Note*: Next Generation Science Standards are taken from NGSS (2013) and College Board learning objectives are taken from The College Board (2015).

## 2 METHODS

### 2.1 Motivation

The goal of yEvo is to involve students in an authentic research experience that connects genetics, cellular and molecular biology, and organism‐level phenotypes in an evolutionary context (Figure 2). As such, yEvo addresses complex and often abstract concepts, such as randomness and temporal scales, with which students frequently struggle (Dougherty et al., 2011; Tibell & Harms, 2017). We developed yEvo in collaboration with teachers at two U.S. schools, a private school in California and a public school in Idaho, to meet the needs of the teachers and their students. The module activities were tailored for the time and resources available to each teacher, which resulted in some differences among the focal classrooms in this study (Figure S1). The modularity of yEvo allows teachers flexibility in including it in their curriculum. As such, we do not prescribe a particular number of yEvo modules. Modules are designed to touch on many topics in a standard biology curriculum (Table 1). Teachers can select modules that best align with their classroom needs. During the five modules, students select yeast mutants that are more resistant to clotrimazole, examine genome sequencing data to identify mutations that may be responsible for this resistance phenotype, and use cellular and molecular models to contextualize how their mutations may be connected to the resistance phenotype. For each module, lab skills, suggested discussion topics, science standard alignments, and example learning objectives can be found in Table 1. Additional information on our design philosophy can be found in Text S1.

### 2.2 Lab module descriptions

We have structured our protocols such that they do not require advanced equipment. The most costly components are sterile test tubes for microbiology work, yeast media, and micropipettes for Modules 1, 3, 4, and 5. Module 2 requires computers with internet access. Material lists for each module can be found in module protocols (Texts S2–S6).

In Module 1 (‘Evolution of Drug Resistance’), students grow yeast in the presence of an over‐the‐counter antifungal azole drug (FungiCure; active ingredient clotrimazole) for several weeks (Figure 2). Students transfer their yeast into new drug‐containing medium at regular intervals (e.g., at the start of one class period each week of the experiment) and increase the drug dosage as they observe improved growth (Text S2). Transfers take ~10–20 min depending on the protocol utilized and the familiarity of students with the procedure. The length and frequency of interaction with these experiments are flexible to classroom time constraints. At the California school, students carried out the experiment for the majority of the school year, transferring yeast to fresh media every class period for up to 34 weeks. At the Idaho school, students transferred their yeast once a week for up to 15 weeks. To reliably observe an increase in clotrimazole resistance, we recommend a minimum of five transfers (~50–75 min of total class time spread over five class sessions), though we were able to isolate clones with increased clotrimazole resistance from as early as two transfers (Taylor et al., 2022). The remaining modules (2–5) allow students to investigate the mechanisms of evolved drug resistance in their yeast from Module 1 or, if they have not completed Module 1, in yeast isolated from prior classrooms that completed this module.

Module 2 explores the genetics of evolution. We have determined the genome sequence of 99 clones of yeast from student experiments and identified mutations that occurred during these experiments. Students use freely‐accessible online databases such as the *Saccharomyces* Genome Database (yeastgenome.org) and NCBI BLAST (https://blast.ncbi.nlm.nih.gov/Blast.cgi) to learn about these mutant genes (Figure 2).

Modules 3 and 4 center around measuring adaptive phenotypes in evolved yeast (Figure 2). Module 3 asks students to inoculate evolved and ancestor yeast into several concentrations of drug. Evolved clones will reliably grow in higher concentrations than the ancestor. Module 4 utilizes a competition experiment in which yeast expressing different pigments are mixed and grown in the presence of clotrimazole and then plated on an agar‐based medium. If one strain in the mixture has a higher fitness, it will produce more viable cells, which can be observed by counting colored colonies on the agar surface.

Module 5 investigates a metabolic trade‐off (Figure 2). Evolved yeasts that are resistant to clotrimazole frequently lose the ability to carry out cellular respiration, which provides a growth advantage in the presence of clotrimazole. However, these resistant mutants cannot grow on a medium containing a non‐fermentable carbon source, demonstrating that some evolved characteristics can be detrimental in the wrong environment (a trade‐off). More details about each module and its protocols can be found in Texts S1–S6 and on our website yEvo.org. For access to data, see our publication on mechanisms of clotrimazole resistance (Taylor et al., 2022).

### 2.3 Teacher and student characteristics

The three focal teachers (referred to here as Liam, Emily, and David) are all experienced teachers who have been in their current positions for over 8 years. All teachers are white, one female and two males. Liam holds a Ph.D. in cellular and molecular biology, and Emily has a Master's in wildlife biology. Liam teaches AP Biology at an all‐female school in California, and Emily and David both teach 10th grade Honors Biology at a mixed‐gender school in Idaho. As part of our proof of concept pre/post surveys (described below), we asked students to respond about their race/ethnicity, age, and primary language spoken at home. Data reported here are from 14 of Liam's students (all female) who responded to our surveys in 2018–2019, and 63 of David and Emily's students (approximately half female, half male) who responded to surveys in 2019–2020 (Table S1). Across both schools, most students spoke English at home; a few students (1–3) under each teacher spoke other languages at home (Table S1). Liam's students were between 15 and 17 years old, and half of his students (7 of 14) identified as multiple races/ethnicities (Table S1). On average, the Idaho students were 1 year younger than Liam's students, and the student population was less diverse in race/ethnicity (Table S1). All California students had taken a previous 9th grade biology course, and about two‐thirds (69%) had previous lab course experience. A small number of California students (3/16 on the 2018–2019 pre‐lab survey) reported having worked in a professional lab or clinic setting. For the Idaho students, this was their first biology‐focused science course, though they had some biology exposure from a 7th grade life science course.

### 2.4 yEvo development and implementation

Modules 1, 2, and 4 were developed and piloted during the California school's 2017–2018 school year (Figure S1). Modules 3 and 5 were developed during the 2018–2019 school year and piloted at the California school (Figure S1). All protocols are based on standard research lab practice but modified in collaboration with classroom teachers to fit their needs. The Module 1 protocol was modified to fit the class structure at the Idaho school by having students transfer yeasts to a new growth medium once per week and carry out the Module 1 experiment for 15 weeks. Detailed protocols can be found in Texts S2–S6. Teachers incorporated lab activities with consultation from researchers but followed their existing classroom plans. In addition to the modules described, some students from these classes participated in other research‐related activities and field trips (e.g., additional experiments, conference presentations, visits to researcher labs) before or after the modules were implemented, though these occurred before or after our surveys were issued.

### 2.5 Survey development

We developed an online survey using questions from two published instruments (Jeffery et al., 2016; Richard et al., 2017) and novel yEvo‐specific questions related to the modules (Text S7). Our goals were to evaluate (1) how students conceptualized topics introduced through yEvo, (2) which aspects of yEvo students liked and disliked, (3) how yEvo impacted students' confidence in their ability to perform scientific investigations, and (4) changes in students' interest in STEM and biology‐related careers. Interview questions were developed to follow up with student responses to survey questions via remote interviews (on Zoom; Text S7). Prior to any module activities, parents/guardians were sent a handout with information about the study and a form to return if they did not consent to share their child's data for the study (passive consent). Students also assented to share their data through either a hardcopy or online form. Teachers responded to interview questions to clarify their classroom activities and capture feedback for future iterative improvements via remote interviews (Text S7). All research methods were submitted and determined to qualify for exempt status by the University of Washington (IRB #00003148).

We also collected the AP Biology test scores from 2014 to 2019 classes at the California school to compare results from classes with and without yEvo modules. We calculated a weighted score for the class using the formula 1/2 (mean multiple‐choice score) + 1/2 (mean free response question score). We additionally calculated a global average for all who took this exam. The values shown are the differences between those scores.

### 2.6 Analysis of student evaluations

Quantitative survey responses were analyzed by comparing the teacher group averages and individual student changes in the pre‐ and post‐survey using *t*‐tests (Table 2). To directly compare pre‐ and post‐changes, we used only paired pre‐post responses for some analyses. Student responses to question 5 (Figure 2) were evaluated on a 0–3 point rubric for correctness and are summarized in Table S2. All codes and the scoring rubric were iteratively developed among three co‐authors (multiple rounds of inter‐rater coding comparisons) and coded blind with regard to student classroom and pre‐post status. For analysis, most Likert‐response questions were converted to a numerical scale where Strongly Disagree = 1, Disagree = 2, Neutral = 3, Agree = 4, and Strongly Agree = 5. Short‐answer survey questions were coded for response themes using qualitative content analysis, primarily a summative approach (Hsieh & Shannon, 2005).

**TABLE 2:** Survey questions, analysis method used, and location of additional information in supplemental tables.

Question	Analysis	Code description	Results
1. What is a gene?	Coded for key terms/concepts and alternative conceptions	Table S3	Table S8; Figure S3
2. What is a mutation?	Coded for key terms/concepts and alternative conceptions	Table S4	Table S9
3. How would you describe evolution?	Coded for key terms/concepts and alternative conceptions	Table S5	Table S10; Figure S3
4. What role do mutations play in evolution?	Coded for key terms/concepts and alternative conceptions	Table S6	Table S11; Figure S3
5. How would you explain antibiotic resistance to a fellow student in this class?	Scored based on correctness (0, 1, 2)	Table S2	Figure 4
6. Individual microbes develop mutations in order to become resistant to an antibiotic and survive.	Compared level of agreement with open‐ended explanation. Coded explanation for key terms/concepts and alternative conceptions.	Table S7	Table 4; Figure S2

*Note*: Additional information on the results of our evaluation program can be found in Text S8.

## 3 RESULTS

### 3.1 Summary of the first 3 years of yEvo

We set out to develop lab‐based modules that would truly allow students to participate in the research process. Over the first 3 years, 203 students from the California and Idaho schools completed Module 1, which provided a high degree of replication and led to our team identifying new genetic factors that contribute to clotrimazole drug resistance in yeast (Taylor et al., 2022). From these Module 1 experiments, we ultimately isolated 99 clotrimazole‐resistant clones, which were subjected to further analysis and whole‐genome sequencing. Every student clone we sequenced possessed at least one mutation that fits known resistance mechanisms to clotrimazole (Taylor et al., 2022). This result demonstrated that our protocol could reliably reproduce clinically relevant findings from traditional research laboratories. In addition, student data suggested previously uncharacterized mechanisms of resistance, providing new insights into clotrimazole resistance (Taylor et al., 2022).

During these first 3 years, we used a combination of surveys and interviews to assess the impact of the program and to identify strengths and weaknesses we could iterate on (Figure S1). A total of 72 students across the California and Idaho schools completed our pre/post surveys. For the remainder of the results, we will move chronologically through our proof of concept evaluation.

### 3.2 Development of yEvo modules in California classes 2017–2018

The first implementations of yEvo were at the California school during the 2017–2018 academic year. Students participated in Modules 1, 2, and 4, which included performing the evolution experiments, analyzing the mutations in the evolved strains, and competing evolved strains to measure fitness (Figure S1). All students were able to obtain clotrimazole‐resistant populations in 7 weeks using the Module 1 protocol (Text S2). Students used their populations in the competition experiment described in Module 4 (Text S5). University collaborators sequenced one clotrimazole‐resistant clone from each student's experiment, and these data were used in Module 2 (Text S3). Results from this sequencing are described in Taylor et al. (2022).

A total of 17 students completed a post‐lab survey. Overall, most students (16/17) stated that they were willing to participate in yEvo again because it was “fun.” One student wrote, “After lots of years of learning science exclusively in a classroom, it was fun to feel like we were doing ‘real’ science and seeing applied concepts. Also, having taken the AP exam, it was a lot more beneficial to have actually done processes than to have just memorized terms.” However, students reported struggling with several aspects of the project, including the amount of time required to perform the evolution experiments (Module 1) and the sequence analysis activities (Module 2). We identified four themes in the positive and negative responses described below. These responses helped us refine the modules to better feature the aspects students enjoyed and learned the most from and improve the presentation of concepts the students found overly confusing.

### 3.3 Theme 1: Agency in running experiments

Most students stated that the process of transferring their yeast to fresh media each class period and watching their yeast grow was a positive experience (11/17 surveyed). For example, one student wrote, “It was fun to do the same thing every day and see our yeast grow and get stronger.” They also enjoyed choosing the dose of clotrimazole to which they exposed their yeast, though sometimes this was frustrating for them when they increased the dose to a level that prevented their yeast from growing and had to go back to an earlier transfer to recover their experiment. These instances provided an opportunity to discuss the limits of their yeast's drug resistance and reflect on how that changed over the course of the Module 1 experiment. Four of the surveyed students expressed that they appreciated mastering the technique of sterile transfers, saying things like, “It became a routine that we got good at, and we were able to practice scientific procedures.” These findings led us to emphasize student agency in the framing of Module 1 in subsequent implementations.

### 3.4 Theme 2: Motivated by competition

Module 4 consists of paired competition experiments to find which evolved strain could grow the best in a high drug concentration. The promise of determining which group's yeast reached the highest fitness in clotrimazole media was a significant motivator for many students (14/17 surveyed), though 3/17 mentioned a dislike of losing. One student wrote, “This part was really fun. I loved competing with the [other] group. It got me really invested in the well‐being of my yeast.” Following this feedback, we now encourage students to explore different strategies for maximizing the fitness of their strains (Text S2).

### 3.5 Theme 3: Scaffolding of competition experiments

Some aspects of the competition experiments, however, were confusing to students, such as the process of counting colonies (2/17). One route to clotrimazole resistance involves loss of cellular respiration (by mutations in the mitochondrial genome; Hallstrom & Moye‐Rowley, 2000). Respiratory deficiency results in a slow‐growth phenotype on media without clotrimazole, a “petite” mutant. These petite colonies were much smaller than their competitors, which was difficult for many students to reconcile with their conceptions of improved fitness in the context of clotrimazole resistance. To address students' confusion in later classes, we introduced the petite phenotype explicitly through the addition of Module 5, which uses this phenotype as an example of the phenomenon of an evolutionary trade‐off: when adapting to one condition leads to decreased fitness in an alternate condition. Additionally, we emphasized that the number of colonies is more important than the size of the colonies. Colony size only reflects growth rate on agar media lacking drug, which is not the phenotype under selection during the experiment (Text S5).

### 3.6 Theme 4: Scaffolding of sequence analysis

One of the most confusing aspects for students was analyzing the DNA sequence data (Module 2); 15/17 mentioned something they disliked about this activity, such as, “It was a little complicated to see some of the mutations” and “It's a lot of letters and it's sort of dizzying.” In some cases, the confusion reflected doing authentic scientific research, such as, “Researching the causes of the effects was difficult because there was no real guide as this is a sort of novel experiment so it was hard to find substantial information.” Some of this confusion likely stemmed from using the “raw” format that standard sequence analysis software returns to users. These mutation files included extraneous information, such as a “quality score” that reflects a statistical measurement of whether a mutation call might be a false positive. We also experienced difficulties implementing a sequence analysis and visualization program, the Integrative Genomics Viewer (IGV), such as long loading times on student laptops. These issues distracted from the goal of Module 2. One student described “having to look at the confusing yeast sequence in the confusing program that did not seem to work on anyone's computer.” In year 2, we simplified the mutation files by removing much of the unnecessary information and focused attention on the literature search component. Students now proceed from a mutant gene name to the *Saccharomyces* Genome Database (yeastgenome.org) to begin exploring gene function (Text S3).

### 3.7 Outcomes from California implementation

During the 2018–2019 school year, California students participated in Modules 1–5, adding activities about measuring resistance to multiple drug concentrations and evolutionary trade‐offs. We collected limited pre–post survey data during this school year (Figure S1), which informed continued iterative improvements of the modules, but we do not report the survey results here.

**FIGURE 3:**
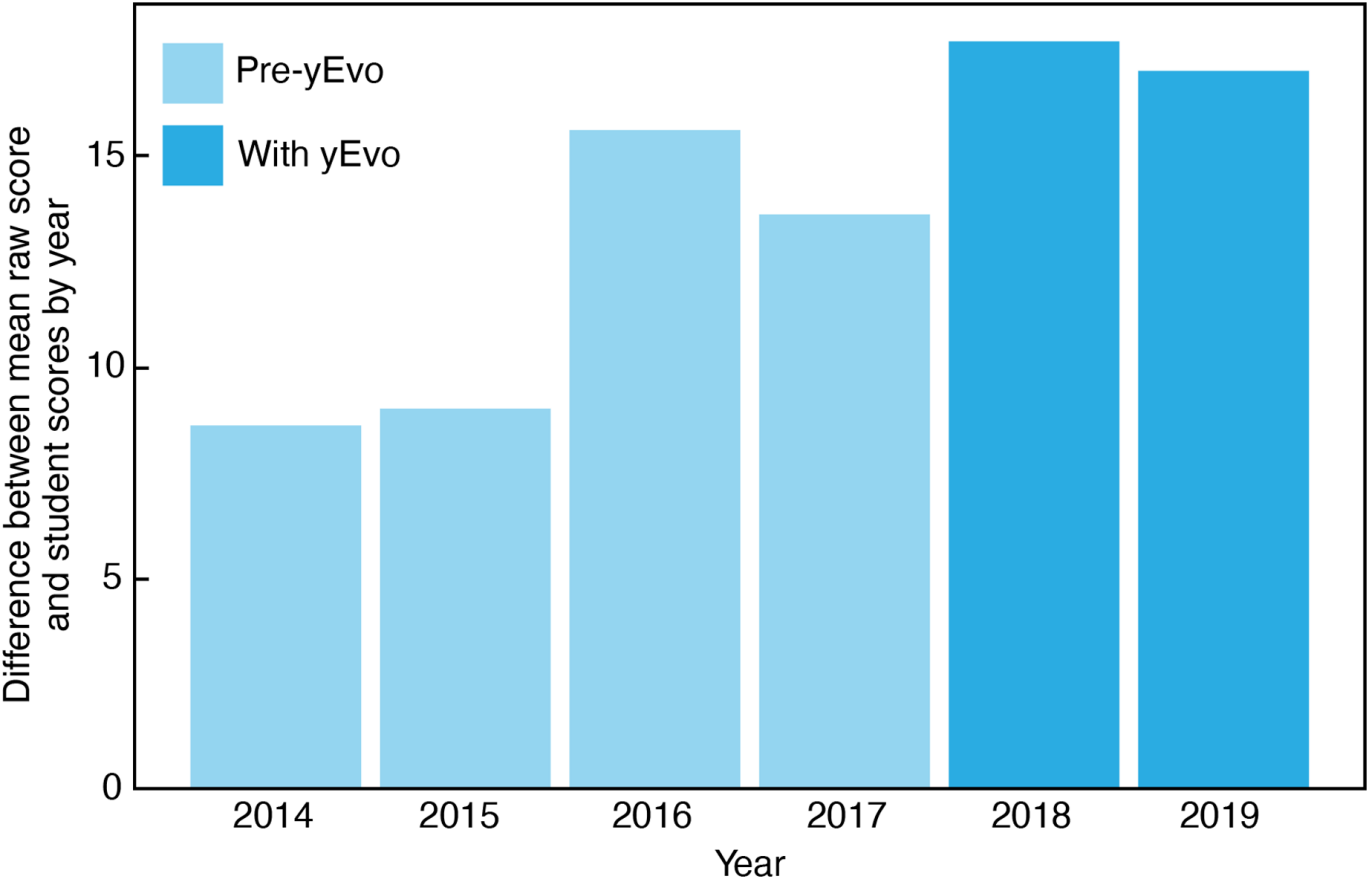
AP scores of California school students over 6 years. Scores were normalized against global scores (Section 2).

To determine how this intensive activity impacted overall learning in the course, we aggregated AP Biology test scores from AP Biology students at the California school from 2014 to 2019: 4 years prior to implementation of yEvo and through 2 years of implementation (Figure 3). Student scores showed a general upward trend throughout this period, possibly reflecting Liam's growing comfort with teaching the material to this population. Scores from the years after implementation continued this general trend and produced the highest scores yet. This result suggests that the significant time allocation for yEvo‐related activities did not detract from student achievement on this standardized test and may have increased proficiency.

### 3.8 Takeaways from Idaho classes 2018–2020

After our initial implementation, we worked with a public high school in Idaho. Students carried out a modified version of Module 1 (experimental evolution) for 15 weeks during the 2018–2020 school years, with transfers reduced to once a week (Section 2). After completion of the experiment, we found that clotrimazole‐resistant yeast were present in student populations in as few as 2 weeks (Taylor et al., 2022). We provided pre‐filled tubes of media with a variety of clotrimazole doses, so students did not have to spend time preparing media prior to transferring their yeast. This reduced the amount of class time required from ~20 min to ~10 min. Most students additionally completed Module 4 (competitions), though this was interrupted by COVID‐19 pandemic‐related shutdowns in 2020 (Figure S1). In the 2019 to 2020 school year, we surveyed 63 students in three classrooms (two teachers) before and after completion of yEvo modules, of which 41 responded to both the pre‐ and post‐survey. When asked about things they liked, responses generally overlapped with those from Liam's class, including references to competition and seeing a change over time (e.g., “I like seeing our yeast evolve to resist, and when we looked at it a week later either being so excited seeing the blue color or being disappointed in seeing the yeast dead”). A majority of students reported that they would be willing to do the activity again (~78%) and that it was fun (~93%) when asked in the post‐lab survey (Table 3). When asked about things they did not like about Module 1, the most common theme in responses (10 students) related to the amount of time required, waiting between transfers, or that the process was slow.

**TABLE 3:** Number of students who responded as agree or strongly agree (“agree”), uncertain, or disagree or strongly disagree (“disagree”) for each of four statements asked in the Idaho post‐survey (combined Emily and David's students, *N* = 41).

Post‐only statements	Agree	Uncertain	Disagree
I would be willing to do this lab activity again because I think it was fun.	32	5	4
I enjoyed participating in this lab.	38	2	1
As a result of participating in this lab, I am more interested in becoming a biologist.	11	14	16
As a result of participating in this lab, I am more interested in pursuing a career in STEM.	13	14	14

### 3.9 Increase in proficiency in an activity‐specific question

**FIGURE 4:**
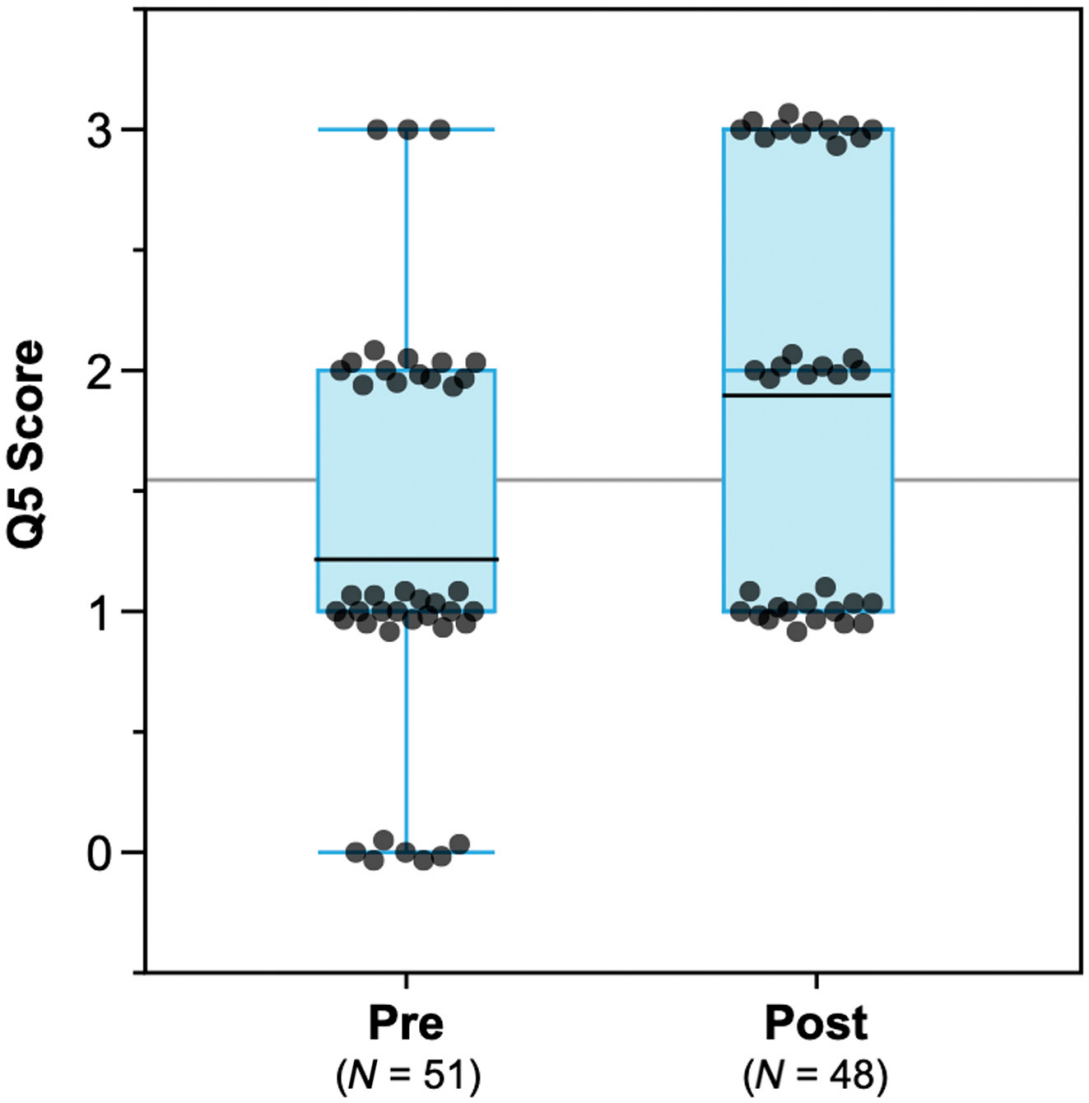
Box plots of question 5 (Q5) scores for the pre (*N* = 51) and post (*N* = 48) responses to the question *How would you explain antibiotic resistance to a fellow student in this class?* Responses on the pre‐ and post‐lab survey were given a score of 0, 1, 2, or 3 based on their correctness (Table S2; Section 2). Points have been distributed vertically and horizontally to reflect the density of responses given a particular score. Average scores for pre and post indicated by a black line; global average gray middle line.

Q5 asked “How would you explain antibiotic resistance to a fellow student in this class?” We found that the average score across both teachers' students of these responses increased by ~55% after participation in yEvo (0.68 out of 3; *p* < 0.0001 on a two‐tailed paired *t*‐test; Figure 4). Though this question asked about antibiotics (general terminology for a drug that is usually associated with bacteria) instead of antifungals, the concepts are nearly identical, suggesting that the students grasped this topic at a conceptual level and could apply it to other contexts.

### 3.10 Response patterns about the mutation process match findings from a previous study

Our Q6 (*[explain why or why not] individual microbes develop mutations in order to become resistant to an antibiotic and survive*) was modeled from Richard et al., 2017, who used it to assess reasoning on mutation processes in evolution in science students and professors. It asked students if they agreed (Likert scale) that individual microbes mutate to become resistant to an antibiotic and then asked them to justify their responses. We coded responses similar to the scheme used in Richard et al. (2017) (Section 2). After completing yEvo, more students disagreed with the statement (12/41 pre vs. 19/41 post; Figure S2). When comparing post‐lab to pre‐lab responses, we saw an overall increase in the use of the codes “individual,” “random,” and “natural selection,” and a decrease in “purpose” (Table 4; Figure S2). Students who agreed with the statement were more likely to describe the mutation as serving a purpose (Figure S2).

**TABLE 4:** Percent of each scored response by teacher, pre and post, with example student responses per term for question 6: [Explain why or why not] individual microbes develop mutations in order to become resistant to an antibiotic and survive. The number of student responses is in parentheses per column (by teacher name, pre/post).

Term	Emily		David		Liam		Example
	% pre (19)	% post (17)	% pre (32)	% post (31)	% pre (13)	% post (6)	
Individual vs. Population (distinguish)	10.5	47.4	31.3	45.2	53.8	50.0	“not individual microbes but colonies” [PP022] “Individual microbes can develop mutations, but those mutations may benefit or hurt them. If it benefits them then with will help the next generation become resistant, but if it hurts them, they will die and it doesn't help any generations.” [PP044]
Random	0.0	15.8	18.8	51.6	61.5	33.3	“They don't develop mutations, but it happens randomly.” [PP005] “First of all, mutations aren't intentionally done.” [PP018]
Purposeful	42.1	26.3	37.5	19.4	15.4	33.3	“Microbes mutate based on the needs of the species, so yes they mutate to be more resistant to antibiotics” [PP099] “they need to mutate in order to fight the antibiotic or else they won't survive.” [PP043]
Natural selection	36.8	57.9	12.5	29.0	53.8	50.0	“If a mutation happens to make the microorganism more resistant to an antibiotic, then it is more likely to survive and reproduce. Over time, most of the population will become resistant to the antibiotic.”

### 3.11 Increase in confidence in the ability to design a valid biological experiment

One of our central goals was to put the tools of science in students' hands. After completing Module 1, 18/46 (39%) students (all collected post‐surveys regardless of pre‐survey completion) reported increased confidence in their ability to design a valid biological experiment, compared to three who listed a decreased confidence (Table 5). In interviews, students expressed that they appreciated learning how to work with yeast and observing how yeast responded to different environments, including statements like, “It was a big excitement every time we see a growth in our tube.” These observations align well with survey responses from students in the California school regarding an appreciation for their newfound mastery of basic microbiology techniques.

**TABLE 5:** Changes in Likert scale questions between pre‐ and post‐lab surveys for David and Emily's students combined.

Difference	I am interested in a career in biology.	I am interested in a career in STEM.	I am confident that I can design a valid biology experiment.	I think about the biology I experience in everyday life.	The study of biology is only useful when it directly benefits human health or well‐being.	Biologists may make different interpretations based on the same observations.	Experiments that are done under lab conditions can provide information that applies to the real world.
‐3	0	0	0	1	0	0	0
‐2	1	0	2	0	3	0	1
‐1	8	7	1	9	5	4	8
0	22	23	23	20	25	27	22
1	8	9	10	6	6	8	6
2	2	1	2	3	0	2	3
3	0	0	3	3	2	0	1
4	0	1	0	0	0	0	0
Mean difference	0.05	0.20	0.44	0.30	0.02	0.20	0.12
Pre‐lab mean	2.85	3.61	3.29	2.93	1.90	4.32	4.00
Post‐lab mean	2.90	3.80	3.73	3.23	1.93	4.51	4.12

*Note*: We converted a Likert scale (strongly disagree—strongly agree) to a numerical 1–5 scale and calculated the difference between paired pre‐ and post‐lab responses for each student who completed both (*N* = 41).

### 3.12 Idaho teacher feedback

After completing the 2019 to 2020 school year, we interviewed the two teachers from the Idaho school about their use of the lab activity. Both teachers had assistance from University of Idaho researchers. They felt that both the lab activity and increased access to trained microbiologists assisted them in teaching aspects of biology that complemented their expertise. As in the student responses, the teachers noted that the competition was a driving factor for student engagement. The teachers liked that the lab gave students freedom in their choices at various steps and allowed students to see evolutionary changes happening quickly. As students gained proficiency with the sterile transferring protocols, they carried out daily or weekly tasks with little assistance in approximately 10–15 min. As a result, these teachers reported that incorporating the lab did not overburden their teaching.

Additional analysis of our evaluation of the Idaho school implementation can be found in Text S8.

## 4 DISCUSSION

### 4.1 Alignment of yEvo goals and outcomes

Here, we outlined the development, implementation, and preliminary evaluation of curricular modules that engage high school students in authentic experimental evolution research. Our design process was greatly aided by close collaborations between experienced teachers and university research labs. This collaboration allowed us to formally and informally monitor how the yEvo lab curriculum impacted student learning of evolution concepts and interest in STEM. We were then able to adjust our approach based on feedback from students and teachers.

We addressed two potential teacher concerns about initiating yEvo in high schools. First, does the amount of time required for these exercises detract from other educational goals? This is a common question we receive from teachers, as standardized test scores are critical for teacher, student, and school evaluation in the US. The California teacher (Liam) co‐developed Module 1. He carried it through the entire school year from 2017 to 2020 and estimated that it took up approximately 20% of total class time. Despite this, participation did not appear to reduce standardized test scores (Figure 3). This result suggests that the benefits of an authentic research project compensated for a reduction in time for traditional content or that teaching some of this content via yEvo was successful. More investigation is needed to determine if this result holds in other classroom contexts.

Our evaluations indicate that students at our second school in Idaho, who used a streamlined protocol (Section 2), improved their grasp of activity‐specific concepts and increased the use of terminology related to several learning objectives. Our findings from Q6 are in line with those of Richard et al. (2017), which showed that undergraduates who disagreed with the statement (“Individual microbes develop mutations in order to become resistant to an antibiotic and survive”) were less likely to display teleological (purposeful) reasoning, a common misconception about evolution. Critically, the use of teleological reasoning decreased after completing Module 1. Students were more likely to disagree with the statement, which the Richard group characterized as an expert‐like response. In conclusion, we found that yEvo, although requiring a significant investment of time by teachers, resulted in a beneficial and authentic research experience for high school students at two different institutions.

Our second concern was: does the repetitiveness of Module 1 impact technical confidence and interest in biology/STEM careers? The length of time for which students carry out these experiments may lead to boredom, and some student responses on the post‐activity survey indicated this. For example, one said, “I feel like the lab had no set end goal, so it sort of just petered out and we lost interest.” We see this process as a worthwhile risk since the repetition allows students to improve their proficiency in the relevant procedures, which is critical to gaining confidence in one's ability. Critically, students, on average, stated that participation in Module 1 contributed to increased confidence in their ability to design biological experiments and an increased interest in Biology/STEM careers (Table 5). Further, students' experiences with these methods may help them better evaluate their interest in a science career and gain essential skills for pursuing a science career. Of students who responded to both the pre‐ and post‐survey, 15/46 (33%) agreed or strongly agreed with one or both questions related to the impact of this activity on their interest in STEM and biology careers (Table 3).

One challenge to evaluating the impact of yEvo, however, is that other classroom activities may have influenced student learning. We also did not require teachers to follow specific learning scaffolds beyond the yEvo laboratory methods. Despite these limitations, we feel that the exploratory work represented in this manuscript demonstrates the value of our research‐education model and lays the groundwork for further investigation.

In addition to these positive signs of student confidence, our educational collaboration was beneficial to the research goals of participating researchers. A large body of research exists on clotrimazole resistance (and azole resistance more broadly) in a variety of species of yeast (Demuyser & Van Dijck, 2019; Gulshan & Moye‐Rowley, 2007). This prior work enabled us to evaluate and contextualize student results. It additionally informed follow‐up work on unexpected findings from module 2, which led to a research publication (Taylor et al., 2022). As a result, future students participating in yEvo will be able to see a lasting impact on the field.

### 4.2 Implementation considerations

The resources required for Modules 1, 3, 4, and 5 are relatively inexpensive and may already be available in classrooms that are outfitted for microbiological work (see Texts S2 and S4–S6), such as test tubes, sterile swabs, and sources of flame. Module 2 requires access to whole‐genome sequencing, which is currently inaccessible for the vast majority of high schools and even many colleges. We have made data from our experiments available so that teachers without access to whole‐genome sequencing can still utilize Module 2. If one has access to sequencing, the sequencing reactions in Module 2 are the costliest aspect of the entire lab (at least $20/sample at the University of Washington lab), a cost the partner labs covered, since these experiments were relevant to ongoing research. As a result, these data serve multiple purposes—education/training and research.

The modules we present cover advanced topics and methods that can be intimidating for teachers that have limited expertise in cell and molecular biology. To develop the modules, we worked with teachers possessing extensive teaching experience and training. The teacher at the California school has a doctorate and one of the Idaho teachers has a master's degree, each in a subdiscipline of biology. In interviews after implementing the evolution module, teachers at the Idaho school reported that the close collaboration with researchers, and the presence of an undergraduate student facilitator at the Idaho school, increased their comfort. Though we did not measure it directly, evidence exists that peer facilitators can benefit students (Sellami et al., 2017). We expect that the interaction with researchers makes the lab feel more authentic than standard classroom exercises. From the researcher's standpoint, the close collaboration gave us more insight into aspects of this project that needed improvement, which we may not have learned without extensive interactions. For those interested in developing similar collaborations, specific suggestions for developing fruitful teacher–researcher collaborations can be found in Warwick et al. (2020) and Knippenberg et al. (2020).

It can be unappealing for researchers to take time away from academic laboratory research to develop educational activities, but our experience (and many others, e.g., Brownell et al., 2015; Jordan et al., 2014; Kerfeld & Simons, 2007; Mavor et al., 2018; Saha et al., 2017) demonstrates that education and research can be merged in mutually beneficial ways.

### 4.3 Future development of yEvo curricula

Initially, each module required completion of Module 1 and ideally of Module 2 for students to participate in Modules 3, 4, and 5. Now, we can provide clotrimazole‐resistant strains evolved from prior iterations of Module 1 and sequencing data from Module 2 to teachers who are interested in later modules. We anticipate that teachers will utilize distinct combinations based on their course objectives, interests, and resources. This process would create natural experiments in implementation through which we would be able to tease out the impact of individual modules by comparing learning gains in classes with or without a particular module. For example, because the Module 1 experimental evolution activity seemed to be polarizing (students reported differences in the aspects that students reported enjoying or not enjoying), it is prudent to investigate how various module combinations or implementation strategies impact overall student engagement and learning. Given our result on increasing student use of terminology in post‐survey responses, capturing how such terminology is used by students and teachers could further help students' conceptual understanding (i.e., first exposure without new technical vocabulary; see McDonnell et al., 2016).

Although students had some agency in parts of the yEvo experiments (e.g., amount of clotrimazole to add in Module 1), they had less agency on the set of questions investigated and overall methodology used. Having such consistent methodology is key to publishable data across classrooms, yet we also want to expand opportunities for student agency in classrooms (Holmes et al., 2020; Vaughn, 2020). To address this aspect, we could incorporate opportunities for students to develop follow‐up experiments or design entirely new experiments based on the experimental evolution paradigm. For example, the Module 1 experimental evolution framework can be applied to any environmental condition that supports yeast growth. This aspect opens the door to framing experiments around applications of yeast biology such as food or biofuel production. However, not all conditions will be equally valuable in a teaching context. The clotrimazole resistance mutations our students isolated have a strong effect, making it possible to see a difference between evolved and ancestral strains after only a few transfers. Prototyping new conditions in the university lab will be a useful precursor to classroom deployment to identify which conditions are most amenable to both teaching and research goals and increase student agency. For instance, our group recently worked with a school to test a new condition (exposure to caffeine). Like clotrimazole, caffeine is a drug that inhibits a specific cellular component. Resistance to caffeine can arise quickly through single mutations in genes that are related to the mechanism of action of caffeine. And like clotrimazole, caffeine is easy to obtain and relatively safe to work with. Results from these experiments can be found in Moresi et al. (2023).

### 4.4 Impact of evolution research experiences

The basic conceptual and technical skillset we aim to impart will be of value for students regardless of whether they continue in biology. Skills related to Module 1, such as sterile technique, liquid handling, and record‐keeping, will be useful in many modern biology applications. Whole‐genome sequencing and related genomic technologies covered in Module 2 are being incorporated into all subdisciplines of biology. Concepts such as the effect of sequence variation on traits, the evolution of drug resistance, and the role of molecular diversity on vaccine efficacy are increasingly making their way into the public sphere. Familiarity with these concepts will certainly benefit public health and understanding of genetic results encountered in health care and direct‐to‐consumer settings. In addition, the collaborative work students used to carry out the experiments is broadly applicable to any career.

We believe that the yEvo experience is a window into the process of evolution that can reinforce concepts throughout high school biology curricula. We designed these modules to illustrate that evolution is an ongoing process that we can study and measure using model organisms with a short generation time. This experience may even provide a route for discussion on evolution with students skeptical of standard evolutionary models. The agency of working with one's own case study in evolution may encourage further learning. Demonstrating how experimental tools are used in modern biology labs may inspire deeper thought into what is currently possible in biological research. These hypotheses will require investigation, and our yEvo framework provides a valuable tool in this endeavor.

## AUTHOR CONTRIBUTIONS

**M. Bryce Taylor:** Conceptualization (equal); data curation (equal); formal analysis (equal); funding acquisition (equal); investigation (equal); methodology (equal); project administration (equal); resources (equal); supervision (equal); visualization (equal); writing – original draft (equal); writing – review and editing (equal). **Alexa R. Warwick:** Conceptualization (equal); data curation (equal); formal analysis (equal); funding acquisition (equal); investigation (equal); methodology (equal); project administration (equal); resources (equal); supervision (equal); visualization (equal); writing – original draft (equal); writing – review and editing (equal). **Ryan Skophammer:** Conceptualization (equal); investigation (equal); methodology (equal); resources (equal); supervision (equal); writing – review and editing (equal). **Josephine M. Boyer:** Conceptualization (equal); formal analysis (equal); investigation (equal); resources (equal); supervision (equal); writing – review and editing (equal). **Renee C. Geck:** Formal analysis (equal); resources (equal); visualization (equal); writing – review and editing (equal). **Kristin Gunkelman:** Data curation (equal); formal analysis (equal); methodology (equal). **Margaux Walson:** Methodology (equal); resources (equal); visualization (equal); writing – review and editing (equal). **Paul A. Rowley:** Conceptualization (equal); data curation (equal); formal analysis (equal); funding acquisition (equal); investigation (equal); methodology (equal); project administration (equal); resources (equal); supervision (equal); visualization (equal); writing – review and editing (equal). **Maitreya J. Dunham:** Conceptualization (equal); data curation (equal); formal analysis (equal); funding acquisition (equal); investigation (equal); methodology (equal); project administration (equal); resources (equal); supervision (equal); visualization (equal); writing – review and editing (equal).

## CONFLICT OF INTEREST STATEMENT

None to declare.

## Supporting information


Data S1.

Table S13.

## Data Availability

The data that supports the findings of this study are available in the supplementary material of this article.
