# Supplementary material for: yEvo: A modular eukaryotic genetics and evolution research experience for high school students
Source: Ecol Evol. 2024 Jan 7;14(1):e10811. doi: 10.1002/ece3.10811 (PMC10771926; doi:10.1002/ece3.10811)
Supplement: Supplementary file 1 — Data S1. [file ECE3-14-e10811-s002.docx]

# **Supplemental Text 1.** Curricular overview.

**Motivation**

The goal of yEvo is to involve students in an authentic research experience that connects genetics, cellular biology, and organism-level phenotypes in an evolutionary context. Briefly, students select for yeast mutants that are more resistant to a “stressor” (any environmental condition that slows growth), examine sequencing data to identify mutations that may be responsible for this resistance phenotype, and use cellular/molecular models to contextualize how their mutations may be connected to the resistance phenotype. These are complex topics with which (in our experience) students frequently struggle. By focusing on **their** experiments and **their** mutations, we expect this experience will provide scaffolding and motivation for further learning.

Experimental evolution can be utilized to study adaptation to any environmental condition that supports the growth of yeast. In our first implementations, we utilized an azole-class antifungal called clotrimazole. Azoles are one of the most commonly used antifungals in medicine and agriculture, and azole resistance is a growing global health crisis. A large body of research exists on azole resistance in a variety of species of yeast, so we and our students can find published information about their mutations to provide context to their results. At the same time, few studies have applied experimental evolution to identify genetic factors contributing to azole resistance, so we felt there would be opportunities for our students to make novel discoveries. We encourage you to consider other experimental evolution conditions and would be happy to support your efforts!

**Module 1: Evolution.** As the ancestral populations for our evolution experiments, we used a collection of yeast strains that were previously engineered to express vibrant pigments by members of the Boeke lab at New York University. This allowed us to watch for contamination of growing cultures by monitoring the color, and was also used for the competition assays in module 4. We added a secondary antibiotic (G418) to reduce the odds of a contamination event and to maintain the plasmids on which the pigment genes are carried.

Students first grew yeast in the presence of an over-the-counter antifungal azole drug (FungiCure) for several weeks, performing transfers into fresh drug-containing medium at regular intervals. As students observed improved growth, they increased the drug dosage. By the end of the evolution experiment, students’ evolved yeast typically grow more robustly in the presence of the antifungal drug due to mutations that increased their level of resistance. Evolved yeast typically survived exposure to much higher concentrations of the antifungal drug (4-16x) than their unevolved ancestors. This result provided an opportunity for qualitative (“In dose X, the evolved culture is ‘cloudier’ than ancestral strain”) or binary (“Both strains grow in dose X but only the evolved strain grows in dose Y”) comparisons. The length and frequency of interaction with these experiments is flexible to classroom time constraints, as resistance phenotypes can be reliably observed after 5 transfers, which can be performed at intervals of 2 days or up to 2 weeks.

Students carried out evolution experiments for 7 to 34 weeks depending on the year and the classroom using one of two protocols. We were able to isolate clones with increased azole resistance from some experiments as early as two weeks.

**Safety.** Baker’s yeast are generally considered nonpathogenic, but isolates have been obtained from hospital patients suffering from complications of compromised immune systems. Drug resistance and pathogenicity are distinct traits, and the laboratory strain we work with in yEvo lacks several characteristics that clinical isolates or pathogenic species possess, such as ability to form biofilms and to grow robustly at human body temperature. Still, it is essential that care be taken in this exercise, particularly around sterilization and disposal of materials.

To prevent contamination by foreign microbes, we recommend utilizing media that can prevent growth of organisms other than the lab strain of *S. cerevisiae*. Our experiments have included a drug called G418, which our particular laboratory strain is resistant to. No method of preventing contamination is perfect, so it is critical to closely observe cultures for signs of contamination. Yeast cells will settle to the bottom of a test tube after 20 minutes in a stationary test tube rack, forming a pellet. Signs of contamination include if the color of the pellet changes, or if cells do not completely settle.

**Module 2: Genomics.** In our trials, teachers sent their evolved yeast to the University of Washington, where we used whole-genome sequencing to identify mutations that may play a role in adaptation. The types of DNA changes represented by these mutations also allowed exploration of the mutational process. Students recovered strains with single base changes, small deletions and insertions, new transposon insertions, and DNA copy number changes ranging from small segments to entire chromosomes. The evolved strains contained mutations in both the nuclear and mitochondrial genome, emphasizing aspects of cell biology present elsewhere in the curriculum.

Students were provided with information about mutations present in evolved yeast that occurred during their or another class’s evolution experiments. They performed a collaborative literature search on mutated genes, and on antifungal resistance broadly, and built hypotheses about how these mutations may impact stress tolerance. This search was aided by the *Saccharomyces* Genome Database (SGD; yeastgenome.org), which curates data and publications from decades of research in yeast. Students used a molecular model of azole drug resistance we have developed to contextualize their mutations.

Yeast can become resistant to azoles through mutations in many cellular processes, but two mechanisms dominate both student experimental results and sequencing of clinical isolates of pathogenic species of yeast. One is through mutations that increase the amount of Erg11 present and thus offset the inhibitory effects of the drug. The second is through mutations that increase expression of membrane proteins called drug efflux pumps that can remove azoles from the intracellular environment. Students were consistently able to identify connections between their mutations and these known mechanisms.

**Modules 3 and 4: Fitness.** The next two modules focused on yeast evolutionary fitness. In the minimum inhibitory concentration (MIC) module, students inoculated their evolved yeast and ancestor into media containing several concentrations of antifungal drug. After 2-7 days, students examined each culture to determine the minimum drug concentration that inhibited growth of each strain. The evolved strain was expected to grow more robustly (as observed by the size of yeast “pellet” at the bottom of a test tube or cloudiness of culture after shaking) than the ancestor in higher azole concentrations.

In the competitive fitness module, students used competition experiments to assess relative fitness of independently evolved strains. Evolved strains of different colors were mixed and grown together in a liquid growth medium with and without azole. The cultures were then diluted and plated onto agar media plates. Each individual cell on this plate will form a colony that expresses each strain’s distinctive color, enabling counting of colonies specific to each strain. A strain that produces more colonies can be said to have a higher competitive fitness than the strain it was mixed with, because more cells from that strain were present in the mixed culture at the end of a period of competitive growth. To frame the activity, we tasked students with identifying the most resistant evolved populations. Student groups were paired in a tournament-style bracket in which the “winner” of each competition moved on to compete their yeast with another winning group.

**Module 5: Fitness Tradeoffs.** Finally, students used a phenotype linked to resistance to visualize a tradeoff in fitness. Evolved yeast have increased tolerance to the antifungal they were selected in, but this antifungal resistance can come at the expense of other traits, leading to decreased ability to grow in alternate environments. In the most extreme example, many antifungal-resistant clones were unable to grow on media in which glycerol is their primary carbon source. Students grew their yeast on an agar plate with dextrose as the carbon source (permissive), picked colonies of yeast, and transferred them to a plate with glycerol as the carbon source (selective). Students recorded the percentage of randomly-chosen colonies that were unable to grow on glycerol media, indicative of a tradeoff between the ability to grow in the presence of an antifungal and the ability to utilize glycerol. The frequency of this phenotype varied widely across experiments and timepoints, another sign of population-level change due to natural selection of new mutations.

**Supplemental Texts 2 – 6. Modules 1– 5 (formatted for classroom use)**

**Azole Resistance Module 1**
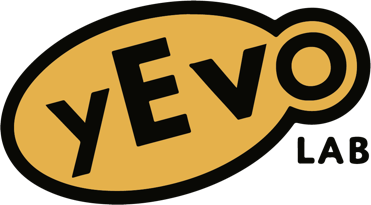


**Evolution of Drug Resistance**

**GOALS**

1. Gain familiarity with sterile technique
2. Master skills of yeast culture by carrying out experiments for an extended period of time
3. Observe evolution in action by seeing the effects of selection pressure on yeast growth

**OVERVIEW**

**Evolution** is change in a population of organisms over time due to natural selection. Evolution acts on a population that has different characteristics, which affect their ability to survive and reproduce in specific conditions, called **selective pressures**. Different characteristics that can be selected upon are the result of mutations, which may exist in the starting population or arise over time. Evolution can be observed and measured using a laboratory technique called **experimental evolution**, in which organisms are grown under defined selective pressures. These can include growing bacteria in the presence of an antibiotic to study mutations that contribute to drug resistance, or growing yeast in acidic media to learn what mutations could improve fermentation of acidic foods. Mutations occur spontaneously, and some rare mutations can provide a fitness advantage. These rare mutations can improve growth under selective pressure, and thus will increase in frequency due to natural selection, sometimes to the point that all surviving organisms in the population contain these mutations.

The budding yeast ***S. cerevisiae*** is an ideal organism to use for experimental evolution because there are many resources available to study its genetics that help scientists interpret the results of experiments. *S. cerevisiae* is also used in many areas beyond the laboratory, where it naturally encounters selective pressures. Yeast used in bread making must be able to grow in high salt environments, and yeast used for industrial processes must be able to obtain energy from diverse fuel sources.

In these experimental evolutions, you will explore how yeast adapts to high concentrations of **clotrimazole**, the active ingredient in the anti-fungal FungiCure. Azole drugs like clotrimazole are one of the most commonly used antifungals in medicine and agriculture, and azole resistance is a growing global health crisis. Using experimental evolution, we can identify genetic factors that contribute to azole resistance. A better understanding of azole resistance can lead to new ways to treat azole-resistant fungal infections.

Every few days, you will transfer yeast in the presence or absence of FungiCure. Without FungiCure, the yeast should always grow well. With FungiCure, growth should be initially poor, but improve over time as mutant yeast arise in the cultures. After you have evolved FungiCure-tolerant yeast, you will compare their fitness with other groups’ yeast to compare their ability to grow in FungiCure (Module 3-4). Some of your yeast will also be frozen and shipped to a university laboratory so they can be sequenced, and determine what mutations arose in your yeast that could contribute to their increased tolerance of caffeine (Module 2).


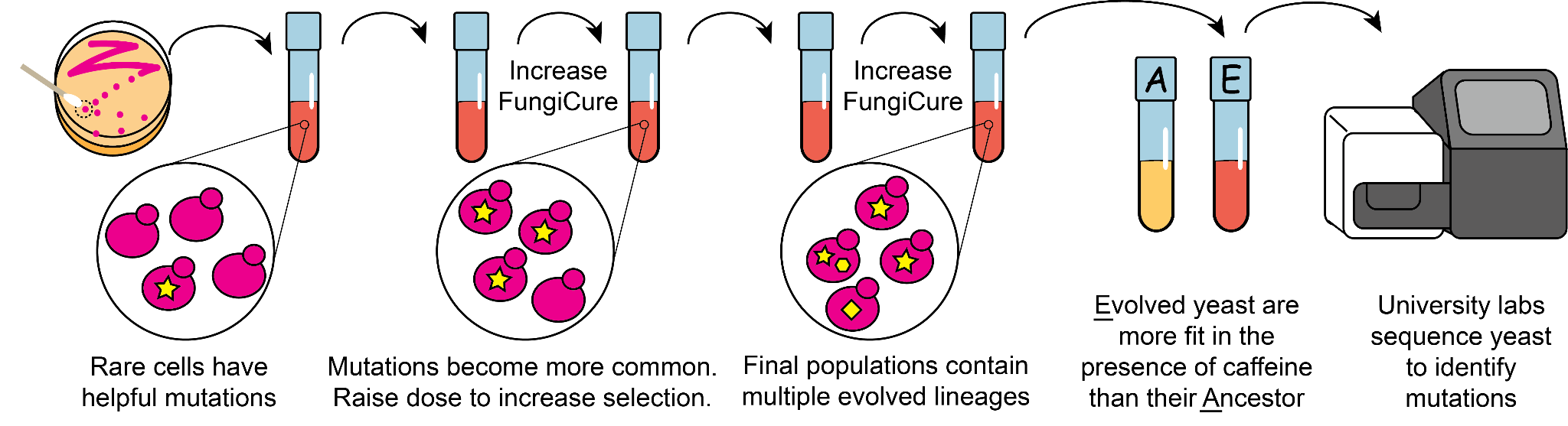


**Figure 1:** **Experimental evolution procedure overview.** *S. cerevisiae* with colored pigments are grown in the presence of FungiCure (clotrimazole). Random mutations arise, and beneficial mutations increase in frequency due to selective pressure as the dose of FungiCure is increased. The final population should be more fit than the ancestor in the presence of FungiCure; those yeast are sent to university labs for sequencing.

**GLOSSARY**

- Clotrimazole: An azole antifungal. Inhibits synthesis of ergosterol, a key membrane component and the fungal equivalent of cholesterol. Clotrimazole is the active ingredient in the FungiCure spray used in this experiment.
- Evolution: Change in a population of organisms over time due to natural selection. Evolution acts on a population that has different characteristics, which affect their ability to survive and reproduce under selective pressures. These different characteristics arise from mutations, which may exist in the starting population or arise over time.
- Fitness: A measure of an individual’s reproductive success in a specific environment.
- G418: Geneticin; an antibiotic commonly used in laboratory experiments. Yeast utilized in this protocol are resistant to G418 due to a plasmid they carry, which also gives them their distinctive color thanks to additional genes on the plasmid that encode pigment production pathways. G418 is necessary for maintenance of the plasmid and additionally helps to prevent contamination.
- *Saccharomyces cerevisiae*: budding yeast, also known as baker’s yeast, a unicellular fungus used in food production, industrial processes, and research.
- Selection pressure: An environmental condition that favors some genotypes in a population over others.
- YPD: A standard rich yeast medium named for its three ingredients: Yeast extract, Peptone, and Dextrose. Also referred to as YEPD.

**TIME ESTIMATE**

When growing yeast in an incubator, we recommend conducting 2-3 transfers per week and carry out the evolution for a minimum of 5 weeks. 5-10 weeks is ideal for the evolution, and allows time for additional modules that measure the fitness of evolved yeast to be carried out after the evolutions are stopped. When growing yeast at room temperature, we recommend conducting 1-2 transfers per week and carry out the evolution for 10-14 weeks.

**MATERIALS AND EQUIPMENT**

Yeast strains

- Ancestral *S. cerevisiae* strains carrying different pigment expression plasmids

Equipment

- Culture tubes (2 per group per transfer)
- P20 and P200 micropipettes and tips (for instructor to make FungiCure dilutions)
- Biohazard waste disposal bin, or a bucket of 10% bleach solution

Consumables

- YPD + G418 liquid media (4mL per group per transfer)
- FungiCure Intensive Maximum Strength Spray (1% clotrimazole in 70% isopropanol)
- Sterile swabs, sterile inoculating loops, or sterile inoculating sticks (2 per group per transfer)

For storage of yeast samples for sequencing (Module 2)

- Cryovials with 0.5mL 50% sterile glycerol (1 per group for every 1-3 weeks)
- Freezer, preferably NOT frost-free (so it does not go through temperature cycles)
- P1000 micropipette or other pipettor to measure 0.5mL
- Fine-tipped permanent marker

*Optional*

- *30^o^C incubator*
- *Test tube roller drum or shaking platform*
- *70% isopropanol (for diluting FungiCure to add directly to students’ tubes)*

**SAFETY**

1. The colored yeast strains are genetically modified and thus considered biohazardous waste. Dispose of inoculating sticks in a biohazard bin, or decontaminate by placing in 10% bleach for 20 minutes before throwing away. Liquid waste containing yeast should be collected and decontaminated using bleach at a final concentration of 10% for 20 minutes before pouring down a drain.
2. **SPILLS:** If there are any spills of the yeast cultures, they should be blotted with paper towels by placing the towels over the spill. Then the towels should be sprayed with 10% bleach and left for 10 minutes before cleaning up.
3. **CONTACT EXPOSURE:** If you spill yeasts on your hands, wash then thoroughly with hot soapy water. If the yeast splash into your eyes, flush them with warm running water. Yeast splashed on clothing should be blotted and washed with soapy water.
4. **CONTAMINATION:** If you notice that the color of your yeast culture has changed or that the culture has gone moldy – **DO NOT OPEN** **the tube**. The tube has become contaminated and the entire tube (including the liquid) should be immersed in 10% bleach. Tubes should be left for 20 minutes before disposal of the liquid down the sink and tubes into the trash or proper glass disposal.
5. **FUNGICURE:** FungiCure Intensive Maximum Strength Spray (1% clotrimazole) is available commercially over the counter. The small quantities used in our experiments **do not** pose a significant health hazard. The FungiCure is dissolved in 70% isopropanol which is extremely **FLAMMABLE**: keep away from flames and other sources of ignition. Isopropanol may cause skin irritation and severe eye **irritation: wear protective eyewear when pipetting FungiCure**. If you spill FungiCure on your hands, wash them thoroughly with warm soapy water. If FungiCure splashes into your eyes, flush them with warm running water.

**BEFORE THE LAB: Week 1**

1. Plan out how the timing of activities will fit with your class schedule. Yeast grow most robustly at 30^o^C. They can be grown at room temperature as well but will grow more slowly. When growing at 30^o^C, transfers can occur 2-3 times per week. When growing at room temperature, transfers can occur 1-2 times per week.
2. Streak ancestral strains with different colored plasmids onto YPD + G418 agar media 3-5 days before the intended start of the lab and place in 30^o^C incubator to grow. If you have a plate with the strains saved, that is sufficient and you can proceed to step 3.
3. Inoculate tubes with different colored strains 1-2 days before the lab. You can prepare one per strain, or one tube for each group. Place 2mL of YPD + G418 media into a tube, use a sterile swab to pick up a colony of yeast, swirl in the media, and put at 30^o^C to grow.
4. Prepare tubes with media. Each group will need 1 tube with 2mL of YPD + G418 (labeled “0µM”) and 1 tube with 2mL of YPD + G418 + 10µM clotrimazole (labeled “2.5µM”).
   1. To make 2.5µM clotrimazole media, add 3.88µL FungiCure (1% clotrimazole) to 50mL YPD + G418 (1:12,800 dilution).

**PROTOCOL: Week 1**

1. Take two two tubes filled with YPD + G418 growth medium, one without FungiCure and one with 2.5µM.

*The negative control is* ***crucial*** *for the students to observe the evolution of resistance. Once the tube with FungiCure appears to be growing as well as the control (and by comparing to their previously recorded pictures or notes), the students should double the concentration of FungiCure.*

1. Label both tubes with your group name and date, and the concentration of FungiCure (“0µM” or “none”, and “2.5µM”)
2. Using a sterile swab, dip it into the yeast culture that you have been provided with.
3. Transfer the damp swab (the liquid in the cotton bud will have millions of yeasts stuck to it) to the tube for no FungiCure.
4. Mix the growth media in the new tube with the swab – remove the swab and dispose of with biohazardous waste (in a biohazard bin or a pot of 10% bleach).
5. Using another sterile swab, dip it into the yeast culture that you have been provided with.
6. Transfer the damp swab to the tube with 2.5µM FungiCure.
7. Mix the growth media in the new tube with the swab – remove the swab and dispose of with biohazardous waste.
8. Record observations about your cultures as instructed in the “Questions: Week 1” section below.
9. Place your tubes in a rack to go into the 30^o^C incubator.

**QUESTIONS: Week 1**

1. As a class, come up with a hypothesis about how the yeast will grow over the course of the evolution experiment. Record that hypothesis.
2. Look at the yeast cultures. You can take a picture and describe what the yeast culture looks like. Is it see-through or opaque (cloudy/muddy)? Compare the tubes that you just inoculated to the tubes that you were given. Write down what you observe.
3. Record your group name and the color of your yeast strain at the top of Table 1. This is important since you will use your name to label your tubes and the yeast that are frozen for sequencing. Make sure that this name is unique and not used by any other groups! (Your instructor may assign you a group name.)

**BEFORE THE LAB: Subsequent weeks**

1. Prepare tubes with media. Each group will need 1 tube with 2mL of YPD + G418 (labeled “0µM”) and 1 tube with 2mL of YPD + G418 + FungiCure. The concentration of FungiCure will depend on the growth students observe. You can prepare tubes with different concentrations of caffeine, or make dilutions of FungiCure in 70% isopropanol that can be directly added to students’ tubes.
   1. For making different concentrations in media, add the µL of FungiCure indicated in the table below to 50mL YPD + G418 media. Scale as needed.
   2. For making different stock solutions (100x concentration), add the µL of FungiCure indicated in the table below to 70% isopropanol to reach a final volume of 500µL. Then add 20µL of 100x FungiCure stock to 2mL YPD + G418 in students’ tubes.

| **Final µM** | **2.25** | **4.5** | **9** | **18** | **36** | **72** | **144** |
| --- | --- | --- | --- | --- | --- | --- | --- |
| µL FungiCure | 3.88 | 7.76 | 15.5 | 31.0 | 62.1 | 124 | 248 |

**PROTOCOL: Subsequent weeks**

1. Retrieve your yeast cultures.
2. Check for contamination: The yeast should have settled to the bottom of your tube, and the media above it remain mostly clear. If the media is cloudy, it could indicate bacterial contamination. Inform your instructor and consider re-starting your culture from a previous week’s tube (or from the original stock if this is the first transfer).
3. Swirl your tubes around so that the yeast are suspended in the liquid.
4. Record observations as instructed in Table 1.
5. Optional: take a picture of your yeast. Look at the photo from last week – notice any changes? Use this information to help you complete Table 1.
6. Decide if you will increase the concentration of FungiCure. If the growth of yeast with FungiCure looks similar to growth of yeast without FungiCure, double the concentration of FungiCure you will use. Record this in Table 1.
7. Transfer the “no FungiCure” culture
   1. Obtain a tube with YPD + G418 media. Label it with group name, date and “0µM”.
   2. Using a sterile swab, dip it into the yeast culture labelled “0µM” that you grew.
   3. Transfer the damp swab into the new tube that you have prepared in step a.
   4. Mix the growth media in the new tube with the swab – remove the swab and dispose of in biohazardous waste.
8. Transfer the “FungiCure” culture.
   1. Obtain a tube with YPD + G418 media. If you decided to increase the concentration, choose the next highest dose of FungiCure, doubling the concentration you used last week. Label it with group name, date and the concentration of FungiCure.
   2. Using a sterile swab, dip it into the yeast culture with FungiCure that you grew.
   3. Transfer the damp swab into the new tube that you have prepared in step a.
   4. Mix the growth media in the new tube with the swab – remove the swab and dispose of in biohazardous waste.
9. Place your new tubes in a rack to go into the 30^o^C incubator.
10. If it is the end of the week, proceed to “Storing yeast for sequencing”
11. Place your old tubes (that you transferred from) into a rack that stays at room temperature. This is in case anything goes wrong with your new cultures, you can return to these tubes instead of starting over from the beginning!

**PROTOCOL: Storing yeast for sequencing**

*This can be completed by each group, or by the instructor / with the instructor’s assistance.*

1. At the end of the week, obtain a cryovial containing 0.5mL 50% glycerol
2. Label the side of the vial with your group name, yeast color, and the date
3. Add 0.5mL of your yeast culture that has been evolved in FungiCure (not the 0µM FungiCure control strain)
4. Invert the tube 5 times to mix together
5. Place the tube in the freezer or collection area as instructed

*These stocks can also be used if a group encounters contamination or loses their sample, and does not have one from a previous week. Use a toothpick or pipette tip to scrape out a bit of the glycerol stock, patch it onto a YPD + G418 agarose plate, spread out, and place in 30*^o^*C incubator for 2-3 days. Pick up multiple colonies with a swab to re-start the culture, to recapture some of the heterogeneity of the frozen population.*

**TABLE 1: Observations and notes - example**

Use this table to record how your yeast is growing in the presence of FungiCure, when you change the concentration, and anything else that occurs during the experiment you need to record.

Example: You have been growing your yeast in 4.5µM FungiCure, and it was of comparable density to the yeast in no FungiCure on 4/3. You decide to transfer it into 9µM FungiCure. When you check your tubes on 4/7, there is no growth in the 9µM FungiCure! You conclude that the yeast was not yet fit enough to survive in 9µM, so you go back to your tube from 4/3 and transfer some to 4.5µM FungiCure to continue to grow. Your notes should look something like this:

| Date | FungiCure concentration | Observations and notes | New FungiCure concentration |
| --- | --- | --- | --- |
| 3/31 | 4.5µM | Culture is very cloudy but not as much as no FungiCure tube. | 4.5µM |
| 4/3 | 4.5µM | Growing like no FungiCure culture! Ready to raise concentration. | 9µM |
| 4/7 | 9µM | No growth. Went back to tube from 4/3 to re-start. | 4.5µM |

**TABLE 1: Observations and notes**

**Group name: _____________________________________ Yeast color: _________________**

| Date | FungiCure concentration | Observations and notes | New FungiCure concentration |
| --- | --- | --- | --- |
|  |  |  |  |
|  |  |  |  |
|  |  |  |  |
|  |  |  |  |
|  |  |  |  |
|  |  |  |  |
| Date | FungiCure concentration | Observations and notes | New FungiCure concentration |
|  |  |  |  |
|  |  |  |  |
|  |  |  |  |
|  |  |  |  |
|  |  |  |  |
|  |  |  |  |
|  |  |  |  |
|  |  |  |  |
|  |  |  |  |
|  |  |  |  |
|  |  |  |  |
|  |  |  |  |
| Date | FungiCure concentration | Observations and notes | New FungiCure concentration |
|  |  |  |  |
|  |  |  |  |
|  |  |  |  |
|  |  |  |  |
|  |  |  |  |
|  |  |  |  |

**QUESTIONS: Final day**

1. What was the highest concentration of FungiCure at which you were able to grow your yeast?
2. Do your results support the hypothesis that you developed as a class in the first week of the experiment? Explain how your data does or does not support the hypothesis.
3. Compare your final concentration and the density (cloudiness) of your evolved yeast culture to other groups. Do you think that your yeast would be more or less capable of growing in a high concentration of FungiCure than other groups’ yeast? Support your answer with observations of the yeast cultures.
4. Where did you encounter challenges in the evolution experiment? Did you ever have to go back to a previous culture? How do you think this affected the fitness of your final evolved yeast in FungiCure? Comparing your experiences and yeast cultures with other groups’ may help you answer this question.
5. If you had time to do another evolution experiment, what condition would you want to expose yeast to?

**Azole Resistance Module 2**
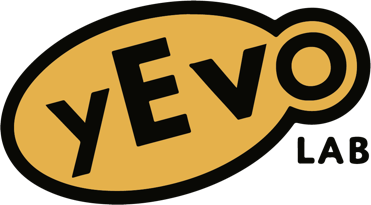


**Genome Sequence Analysis**

**GOALS**

1. Learn how to use online genetics analysis tools.
2. Identify different types of mutations and their effects.
3. Form specific hypotheses on how mutations may contribute to altered fitness.

**OVERVIEW**

The evolution experiments in Module 1 select for yeast mutants that are more resistant to clotrimazole (the active ingredient in FungiCure). These mutants will possess a few specific changes to their genetic code that cause them to be resistant to clotrimazole. After Module 1, we isolated yeast from each experiment and sequenced their genomes to identify their mutations.

In this module, you will receive the sequence of one of the genes found to be mutated in your evolved yeast. Using online tools, you will identify the gene and type of mutation, and research its function in yeast. From this information, you will form hypotheses about how this mutation may contribute to the azole resistance of your evolve strain.

**MATERIALS AND EQUIPMENT**

- Computer and internet access for each participant
- .xlsx mutation file provided by university lab partner, which contains a list of mutations from a strain evolved in Module 1

**INTRODUCTION**

Your yeast have had the genetic code of their entire genome determined! The yeast genome is 12 million letters long. Your yeast likely had between 1 and a handful of single changes to its genome sequence. We have sent you a list of mutations that your yeast possessed, as well as an altered sequence from your yeast. We will walk through how to build hypotheses about what these mutated genes are doing.

Yeast have thousands of genes that each are a blueprint for making a bit of cellular machinery called a protein. These machines come together in intricate ways to form assembly lines (like the ergosterol “pathway”), rigid support structures (like actin), export systems (like the pump *PDR5*), or master regulators of specific processes (like the transcription factor *UPC2*). The goal of this activity is to think through how the DNA changes in your evolved yeast will impact your genes’ protein and ultimately make your yeast more resistant to the FungiCure. Look at **Figure 1** for some examples of genes and pathways that are known to be affected by FungiCure (clotrimazole is the active ingredient in FungiCure).

| 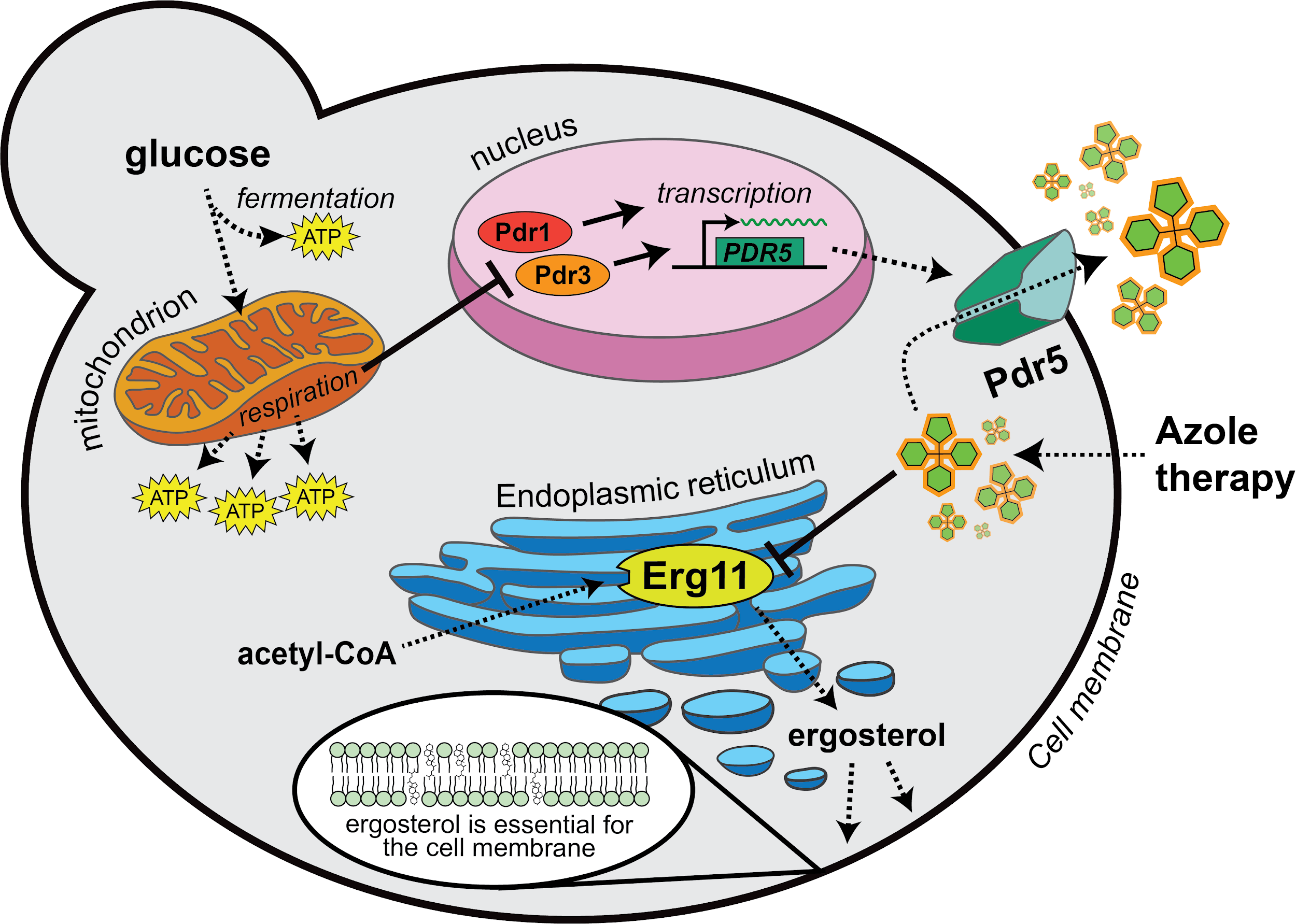 |
| --- |
| **Figure 1:** Pathways affected by azole therapy clotrimazole, the active ingredient in FungiCure. Clotrimazole inhibits the activity of Erg11 to reduce the production of ergosterol, an essential membrane sterol. Proteins and processes that are important for clotrimazole resistance are illustrated. Mutations in genes *PDR1* and *PDR3* or loss of mitochondrial respiration result in an increase in the expression of *PDR5* and the active removal of clotrimazole from the cell. Duplications of *ERG11* compensate for inhibition by clotrimazole. Other pathways not featured here can allow yeast to survive with less ergosterol. |

Now we will use this information to learn more about how your yeast evolved! You have a file named mystery_gene#.fas (with some number in place of the #) that contains the sequence of a gene from your yeast that we think might have been important for this resistance. We will work through the steps to determine what this mystery gene is and its function. We will begin by looking at the DNA sequence of your evolved yeast to see how it has changed, and how this will change the gene’s protein sequence. We will then use a database to figure out what your gene does. This information can be used to understand how your yeast evolved to become resistant to FungiCure.

For a refresher on how a gene’s DNA sequence is used to produce a protein, we recommend checking out [Khan Academy’s Central Dogma section](https://www.khanacademy.org/science/biology/gene-expression-central-dogma), particularly the article on [the genetic code](https://www.khanacademy.org/science/biology/gene-expression-central-dogma/central-dogma-transcription/a/the-genetic-code-discovery-and-properties?modal=1).

**TASK 1: Identify your mutation**

Before we begin our analysis, let’s look at your mutation file.

Your mutation is one of 4 types of genetic changes:

1. **Synonymous mutations** are DNA changes that do not change an amino acid. You will not be able to see these in the protein alignment. They are sometimes referred to as silent mutations because they do not impact a gene’s protein.
2. **Missense mutations** change an amino acid in a gene’s protein. They may change the function of that protein.
3. A **nonsense mutation** results in an early stop in the gene. You will notice the * in the sequence of your query if you have a nonsense mutation. None of the amino acids that come after this stop will be added to the protein, so the resulting protein will be shorter than the original subject (sbjct) version.
4. A **frameshift mutation** is when one or a few nucleotides are inserted or deleted and results in a change in the reading frame of a protein, shifting how the code of the gene is read. Nearly all the amino acids after the mutation will be changed, and this frequently leads to early stops, like in the example blastx alignment above.

**TASK 1 QUESTIONS**

1. What change(s) can you see in your evolved yeast’s DNA?
2. Record how your mutation impacted your amino acid sequence. What was the original codon, and what is the codon after the mutation (see Resource 1). What was the original amino acid, and what is the amino acid after the mutation occurred?
3. Which of the 4 types of mutations (above) matches the one you see? Do you think it will severely affect the function of the protein? Why or why not?

**TASK 2: Learn about the gene affected by your mutation**

We will now move onto the *Saccharomyces* Genome Database (SGD) website to learn more about our genes. SGD contains information from thousands of published studies on yeast, which has been curated to make it easier to find information about a specific gene.

<https://www.yeastgenome.org/>

For this example, I’m going to show you the page for a gene called *ERG11*. This gene encodes instructions for making an enzyme that plays a role in making ergosterol. Ergosterol is like cholesterol, and it helps cell membranes to stay structurally sound. Clotrimazole, the drug you worked with in Module 1, stops this enzyme from working, which ultimately harms the cell’s membrane and prevents it from growing.


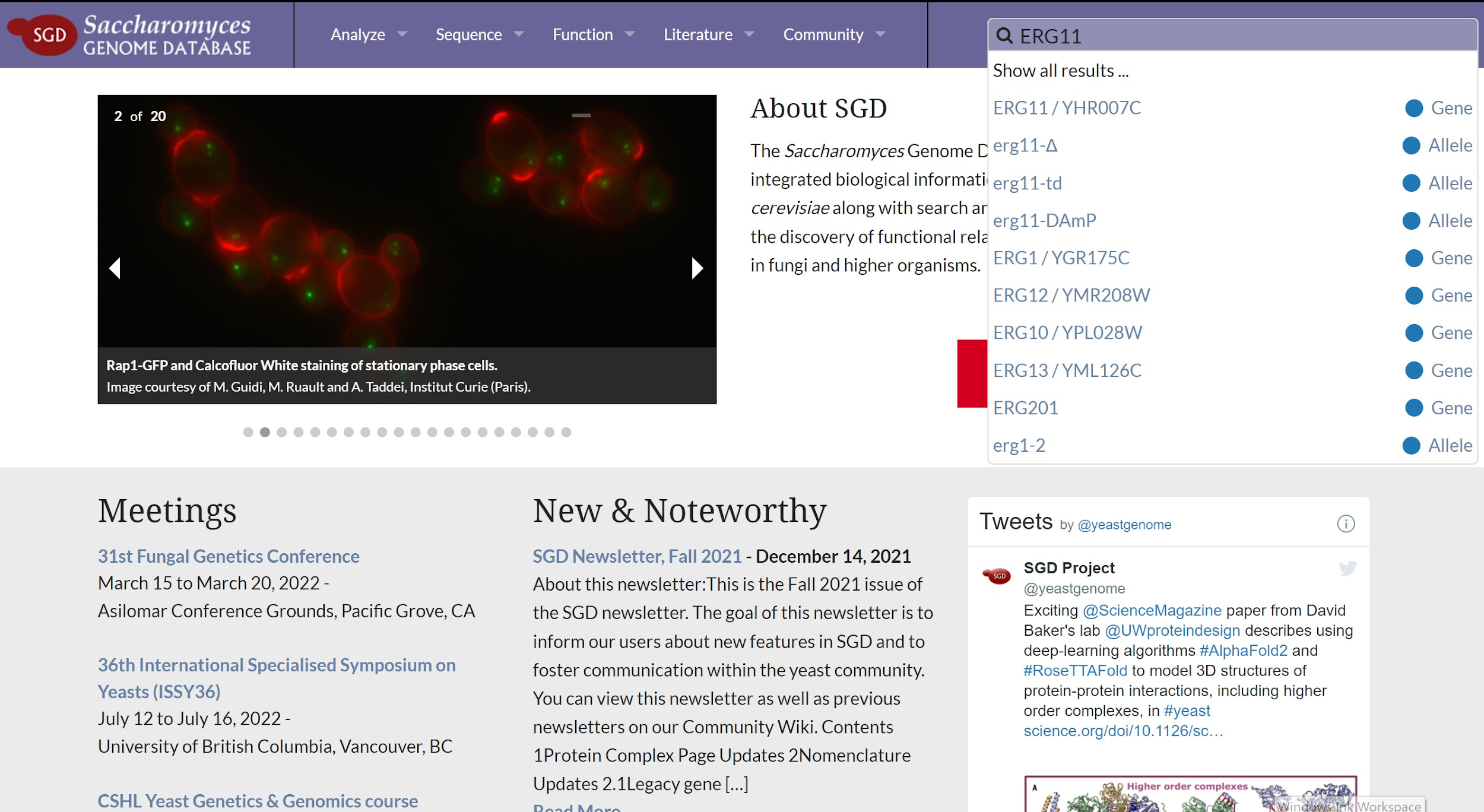


First let’s go to the *ERG11* page. You can do this by doing a google search for “ERG11 SGD”, or by entering “ERG11” in the search bar at the top right of the SGD homepage as shown above.


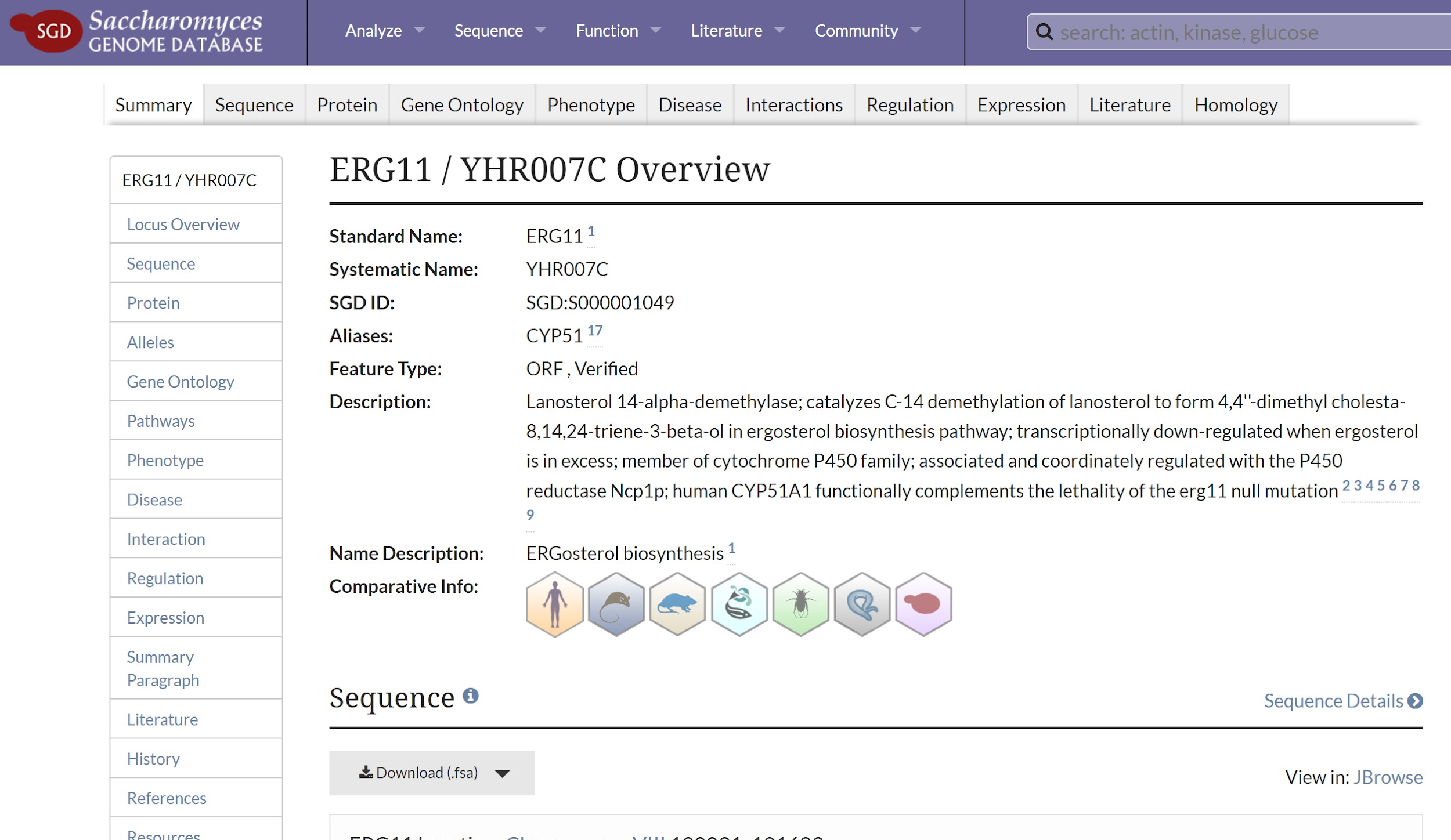


This page contains a ton of info organized into sections that are listed in light blue along the left side of the page. The sections I like to use are *Locus Overview*, *Summary Paragraph*, and *References*. The others are great, but for now you can ignore them.

When you first enter the page you should see the Locus Overview, displayed as “ERG11 / YHR007C Overview”. The Description section will give you a short description of what is known about the gene. It’s extremely dense, and you will encounter a lot of unfamiliar terminology. The trick is to look through for terms that you recognize that are related to the clotrimazole drug from Module 1.

In this case, the term “ergosterol” is a great clue. In Module 1 we worked with a drug that inhibits ergosterol synthesis, and here we have a gene that is important for synthesizing ergosterol. The chances of a gene with this role being randomly mutated in your strain is very low, so there’s a good chance this mutation was selected during the evolution experiment.

The Comparative Info section can be cool to click through. It shows icons that represent other organisms that possess this gene. Most of the genes that make ergosterol in yeast also make cholesterol in mammals, so this section includes an icon for humans, mice, and rats. If you click on the icon you’ll be taken to a different database that has information about how this gene works in those organisms. You don’t need to do that now, but it’s worth checking out if you finish early.

Now navigate down to Summary Paragraph.


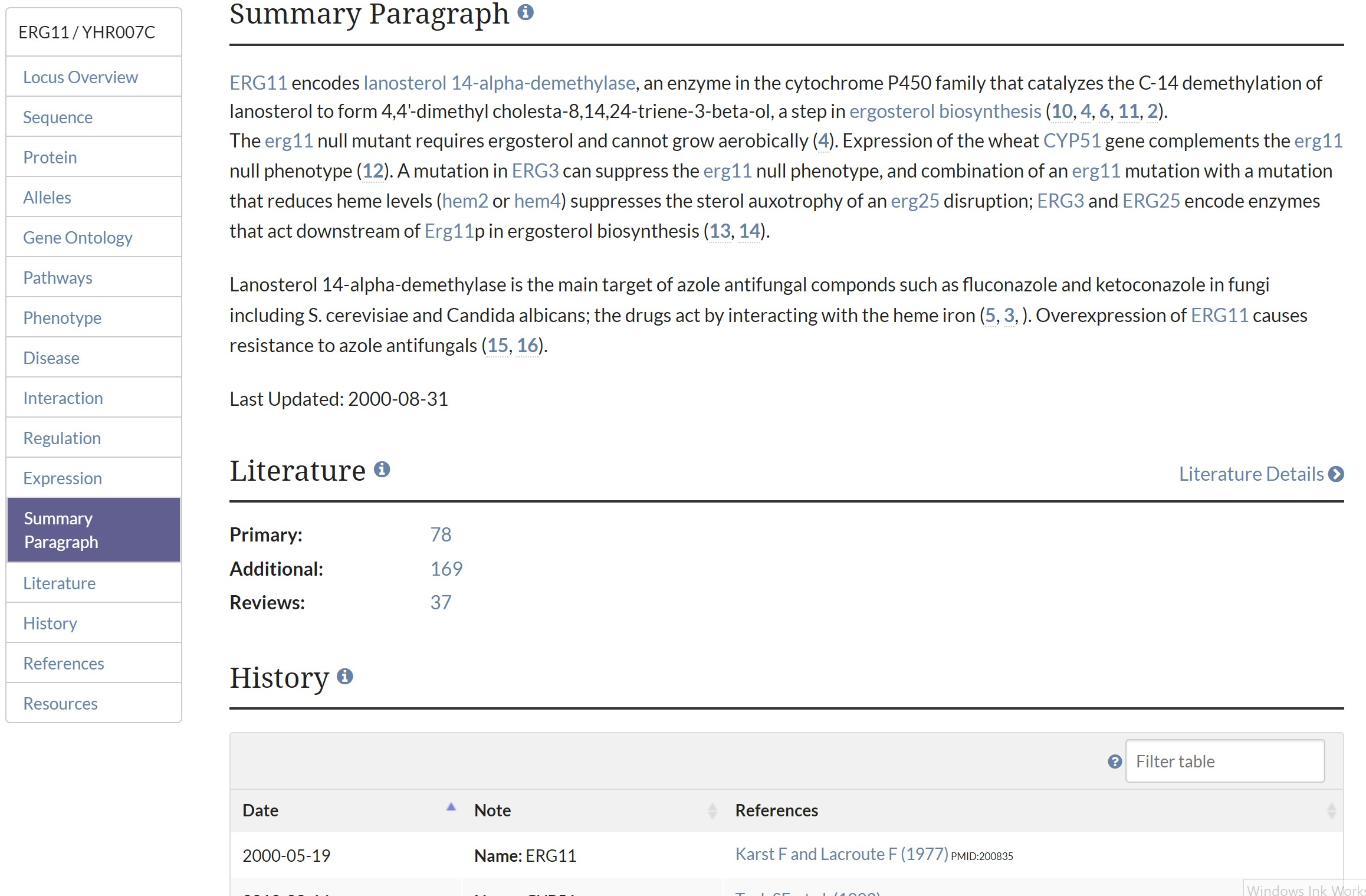
The summary paragraph is a longer description of what we know about this gene. It’s usually in more of a narrative format and can be easier to read (but not always).

Finally, check out References.

**
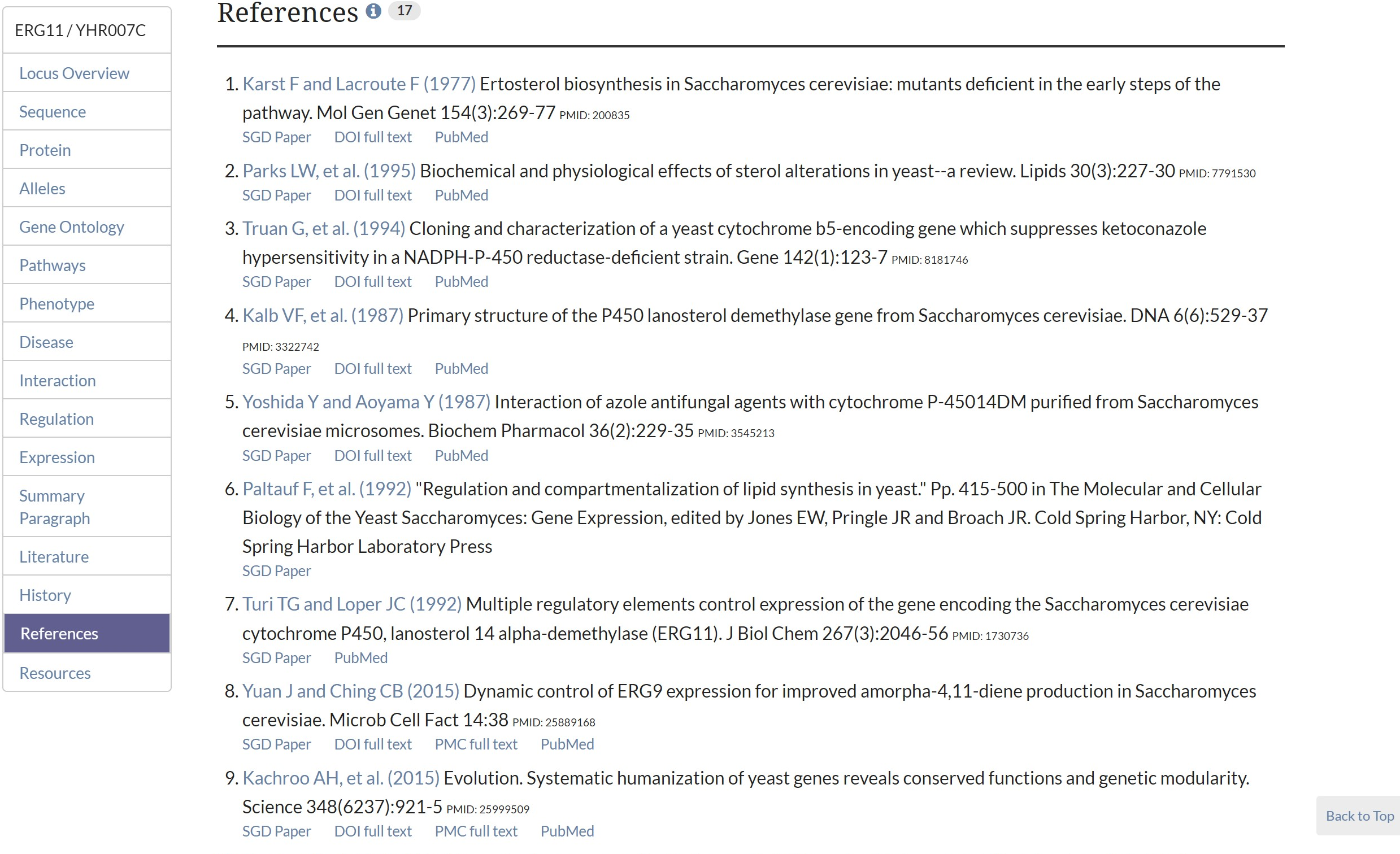
**

This section lists scientific publications that include information about the gene. The SGD page for each gene is made by combing through publications like these to find useful information about what the gene does.

You may have noticed light blue numbers in the sections above. These numbers are citations for information and correspond to publication in this list. If you saw information above that seemed useful, you can click on the “SGD Paper” link under a publication to find a freely-accessible text of the article.

Skimming publication titles in the references section may lead you to more information about what your gene does.

Now, go to the SGD page for one of the genes in your list and answer the questions below.

**TASK 2 QUESTIONS**

1. Find one fact about your gene and record it.
2. What is the function of your gene?
3. Did you notice any keywords related to clotrimazole for your gene?
4. Can you think of a way your gene’s function could be related to azole drug resistance?

**RESOURCE 1**

**Codon table**
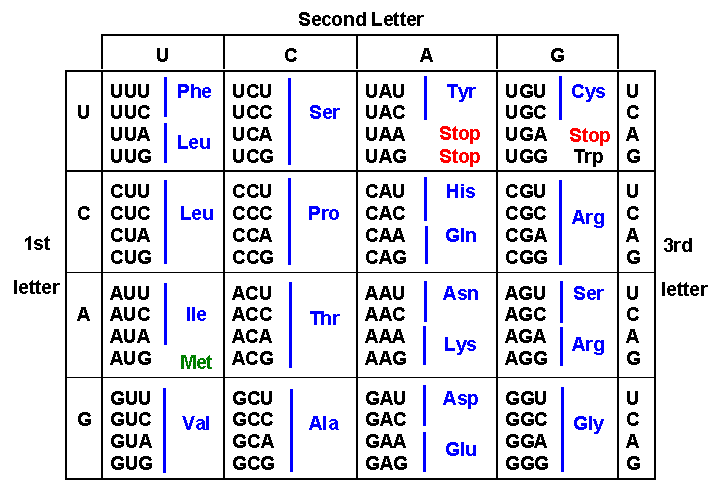


**RESOURCE 2**

**Amino acid codes table**
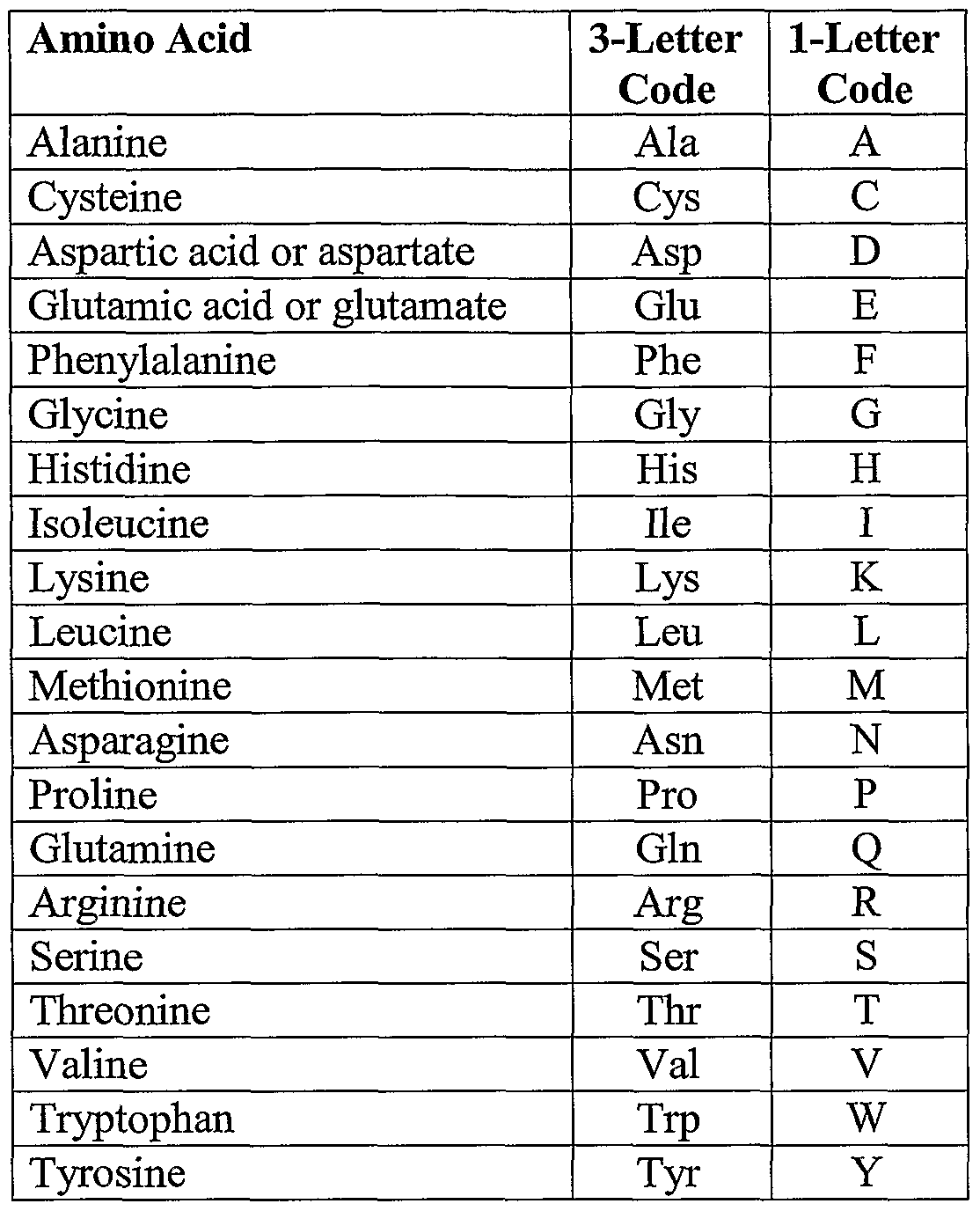


**Azole Resistance Module 3**
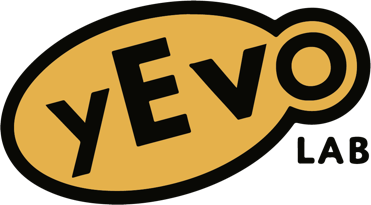


**Fitness – Growth Inhibition**

**GOALS**

1. Demonstrate that strains evolved in the presence of an antifungal are more fit than their ancestors in that environment
2. Demonstrate that independent evolved strains may differ in their fitness in the evolved environment

**OVERVIEW**

The goal of this lab is to provide visual evidence of evolution. You will use a Minimum Inhibitory Concentration (MIC) assay to quantitatively determine the resistance of evolved yeast from Module 1.

In an MIC assay, a microbe is exposed to several doses of a drug to determine the minimum dose that completely inhibits growth. In this instance, we will expose yeast from the Module 1 evolution experiments to several doses of FungiCure. The MIC is the lowest dose of a compound - here, FungiCure - that completely inhibits growth of your yeast strain (**Figure 1**). Yeast that evolved in the presence of FungiCure should be able to withstand higher doses than their ancestors, and therefore have a higher MIC. Not all experiments reach the same fitness, so results may vary!


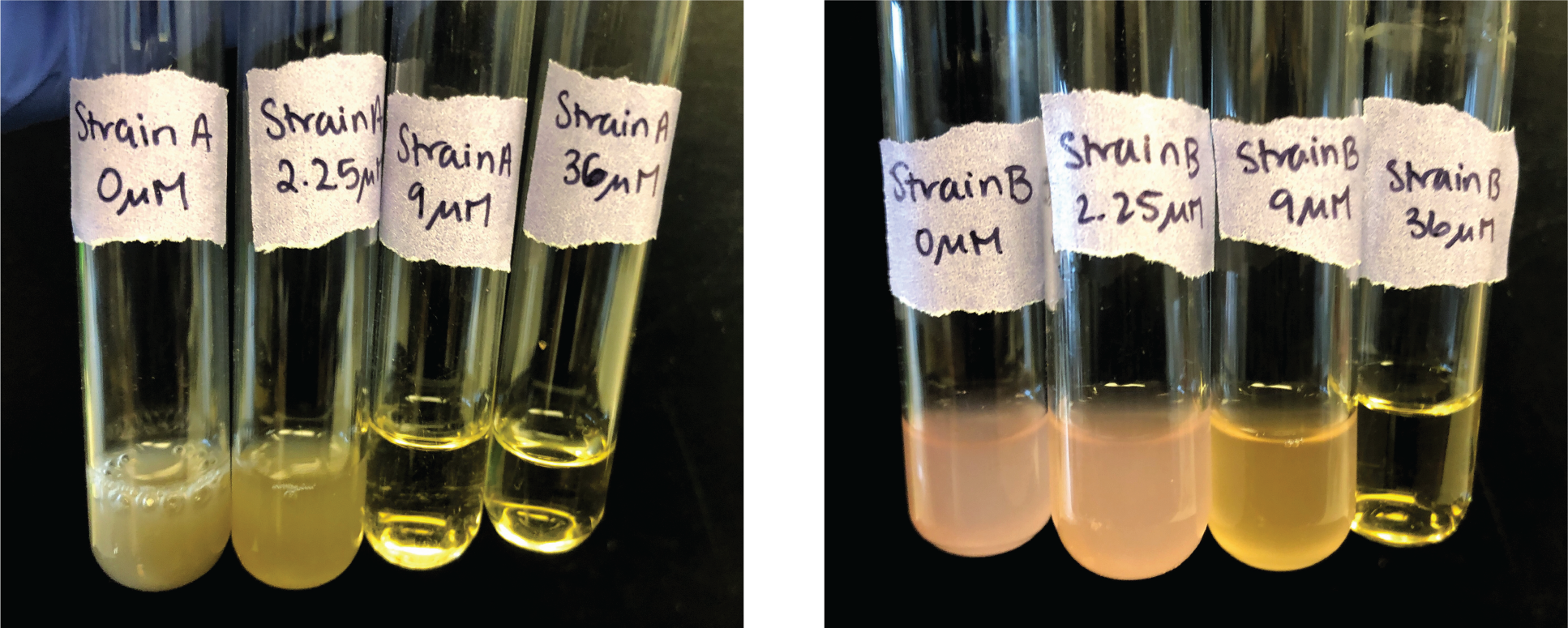


**Figure 1: Minimum Inhibitory Concentration (MIC).** Two yeast strains (A, with a gray pigment, and B, with a pink pigment) were evolved in the presence of FungiCure. After the end of the evolution experiment, a colony from each strain was grown overnight and then inoculated into YPD + G418 media containing different doses of FungiCure (clotrimazole). The lowest doses that completely inhibited growth for A and B were 9µM and 36µM, respectively. Therefore, the MIC of clotrimazole for Strain A is 9µM, and for Strain B is 36µM.

**GLOSSARY**

- Clotrimazole: An azole antifungal. Inhibits synthesis of ergosterol, a key membrane component and the fungal equivalent of cholesterol. Clotrimazole is the active ingredient in the FungiCure spray used in this experiment.
- Fitness: A measure of an individual’s reproductive success in a specific environment.
- G418: Geneticin; an antibiotic commonly used in laboratory experiments. Yeast utilized in this protocol are resistant to G418 due to a plasmid they carry, which also gives them their distinctive color thanks to additional genes on the plasmid that encode pigment production pathways. G418 is necessary for maintenance of the plasmid and additionally helps to prevent contamination.
- MIC: Minimum Inhibitory Concentration. The lowest dose of a drug or other stressor that completely inhibits the growth of a microbe.
- Selection pressure: An environmental condition that favors some genotypes in a population over others.
- YPD: A standard rich yeast medium named for its three ingredients: Yeast extract, Peptone, and Dextrose. Also referred to as YEPD.

**MATERIALS AND EQUIPMENT**

Yeast strains

- Evolved and ancestral *S. cerevisiae* strains (from Module 1)

Equipment

- Pipettes: volume needs will vary based on implementation. You will likely need a P2-20ul, a P20-200ul, and a P200-1000ul or equivalent, as well as a 5ml serological pipette.
- Culture tubes
- Glass beads or plate spreader

Consumables

- YPD + G418 liquid media (at least 30ml per yeast strain per student)
- Sterile swabs, sterile inoculating loops, or sterile inoculating sticks
- YPD + G418 agar plate (to streak out starting strains)

Chemicals

- FungiCure spray with active ingredient clotrimazole

*Optional*

- *30^o^C incubator*
- *Test tube roller drum or shaking platform*
- *Vortex machine*

**BEFORE THE LAB**

1. Plan out how the timing of activities will fit with your class schedule. Yeast grow most robustly at 30^o^C. They can be grown at room temperature as well but will grow more slowly. *We’ve included estimates for the time it’ll take for your students’ yeast to grow where applicable in italics*.
2. Make FungiCure media. Each competition performed will require 5ml each of a low and a high dose of FungiCure media. In our hands, the concentrations below work well, but we encourage you to experiment with additional doses as time and resources permit!

- Low dose: 1:12,800x, 2.25µM (3.88ul of fungicure in 50ml of YPD + G418) inhibits growth the ancestral strains and was the starting concentration for the evolutions.
- High dose: 1:3200x, 9µM (15.5ul of fungicure in 50ml of YPD + G418) prevents growth of the ancestral strains but not the evolved strains.
- Very high dose: 1:800x, 36µM (62.1ul in 50ml of YPD + G418) prevents growth of the ancestral strains and several evolved strains, but some evolved strains will grow.

1. Streak evolved and ancestral strains onto YPD + G418 agar media at least 2 days before the intended start of the lab.

**PROTOCOL**

**Day 1:** Inoculate evolved and ancestral strains of yeast into separate tubes of medium.

1. Fill two test tubes with 5ml each of liquid YPD + G418. Label one “evolved” and the other “ancestor”.
2. Use a sterile swab, inoculating loop, or inoculating stick to pick a colony of either evolved or ancestral yeast and inoculate it into its respective test tube.
3. Allow the yeast in these tubes to grow until you can no longer see through the liquid media. *When growing at 30^o^C in a roller drum or shaking platform, this will take 1-2 days. When growing on a bench top at room temperature without shaking or rolling it will take 2-3 days. Yeast can be left longer than these amounts of time (up to a week) without worry*.

**Day 2:** Mix evolved and ancestral strains in media with or without FungiCure.

1. Fill one test tube each with 5ml of the following three media: YPD + G418; YPD + G418 + low dose of FungiCure; YPD + G418 + high dose of FungiCure; YPD + G418 + very high dose of FungiCure. Label these tubes with the media type used.
2. Examine the cultures you inoculated on Day 1. If yeast have settled at the bottom of the tube (pelleted), gently shake the tube until they are completely resuspended. The liquid sample of yeast (culture) should be dense enough that you cannot see through it, and the two cultures should be comparable in density.
3. **For each strain to be assayed:** Add 5ul of culture to each of the four test tubes you inoculated. *Incubate these as on Day 1.*

**Day 3:** Observe growth of yeast strains in various doses of FungiCure.

1. Examine the cultures you inoculated on Day 2. If yeast have settled at the bottom of the tube (pelleted), gently shake the tube until they are completely resuspended. The liquid sample of yeast (culture) should be dense enough that you cannot see through it, though the culture grown in the highest dose of FungiCure may appear less dense than the others.
2. Record the lowest dose that completely inhibits growth of each of your yeast strains. This is the MIC for that strain.

**EXTENSION QUESTIONS**

1. How do your results compare to those of other groups?
2. Do all yeast adapted to the same environment (evolved to survive in the same dose of FungiCure) have the same fitness in that environment?

**Azole Resistance Module 4**
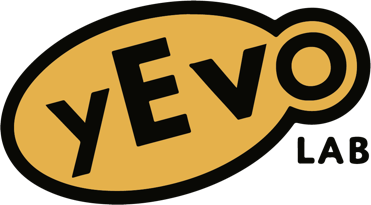


**Fitness – Competition**

**GOALS**

1. Demonstrate that strains evolved in the presence of an antifungal are more fit than their ancestors in that environment
2. Demonstrate that independent evolved strains may differ in their fitness in the evolved environment

**OVERVIEW**

We have been carrying out experimental evolutions with lab strains of *S. cerevisiae* that express vibrant pigments and thus each have a distinct color. Because of these colors, relative abundance of each strain in a mixed culture can be determined by counting colony forming units (CFUs) and calculating the ratio of colors (**Figure 1**). This approach can be used to determine whether yeast with different colors are better adapted to a particular environment.


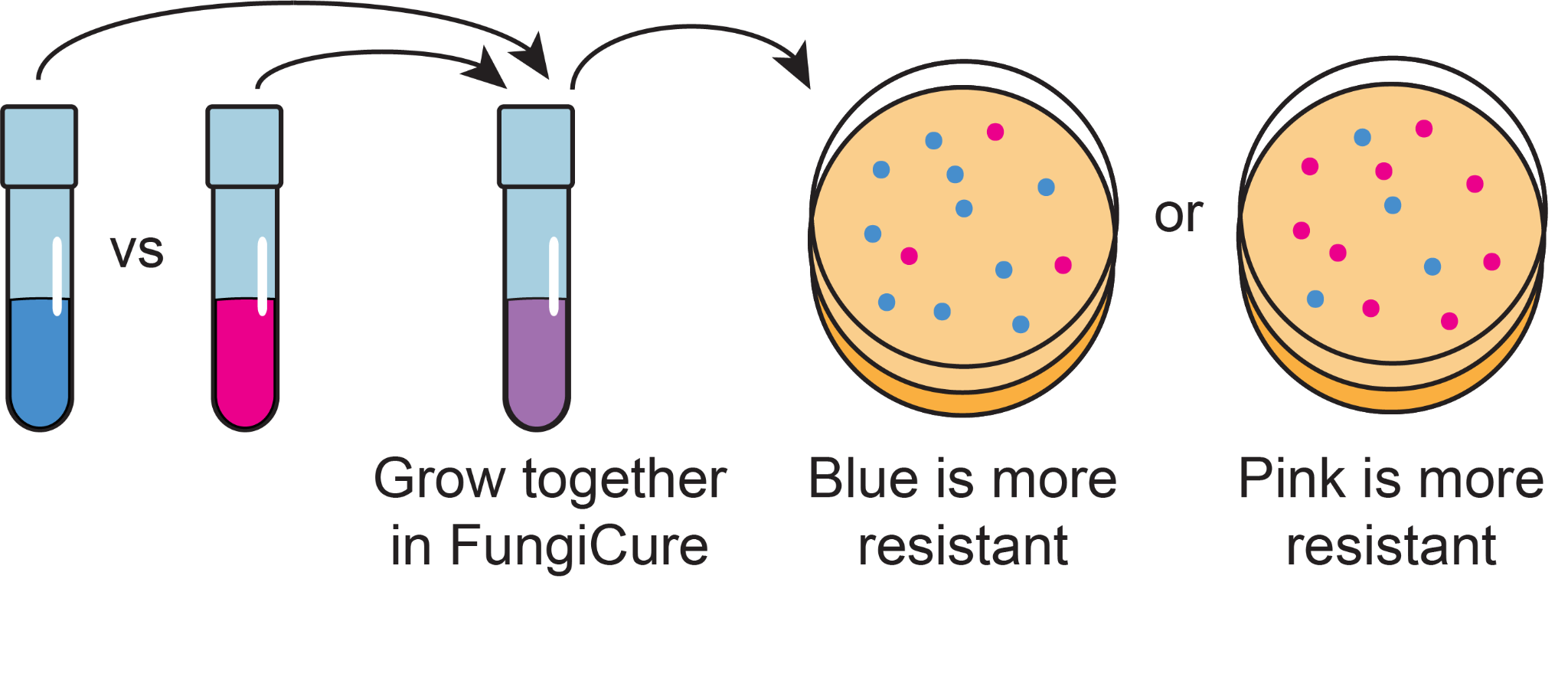


**Figure 1:** **Competition experiment overview.** Ratio of CFU colors on agar media indicates the relative abundance of each strain in a mixed culture.

We will use these colors in a competition experiment, which will allow us to determine which yeast from your experiments are best-adapted to the antifungal drug used in Module 1. To do this, you will first grow yeast from different timepoints in the evolution experiment and with different colors in a medium that does not contain the antifungal. You will then mix these strains in media containing varying concentrations of the antifungal so that they will compete for resources, You will then plate these mixed cultures onto agar media. After a few days of growth, you can count the ratio of colors on each plate to determine which strain “won” the competition in each concentration (**Figure 2**).


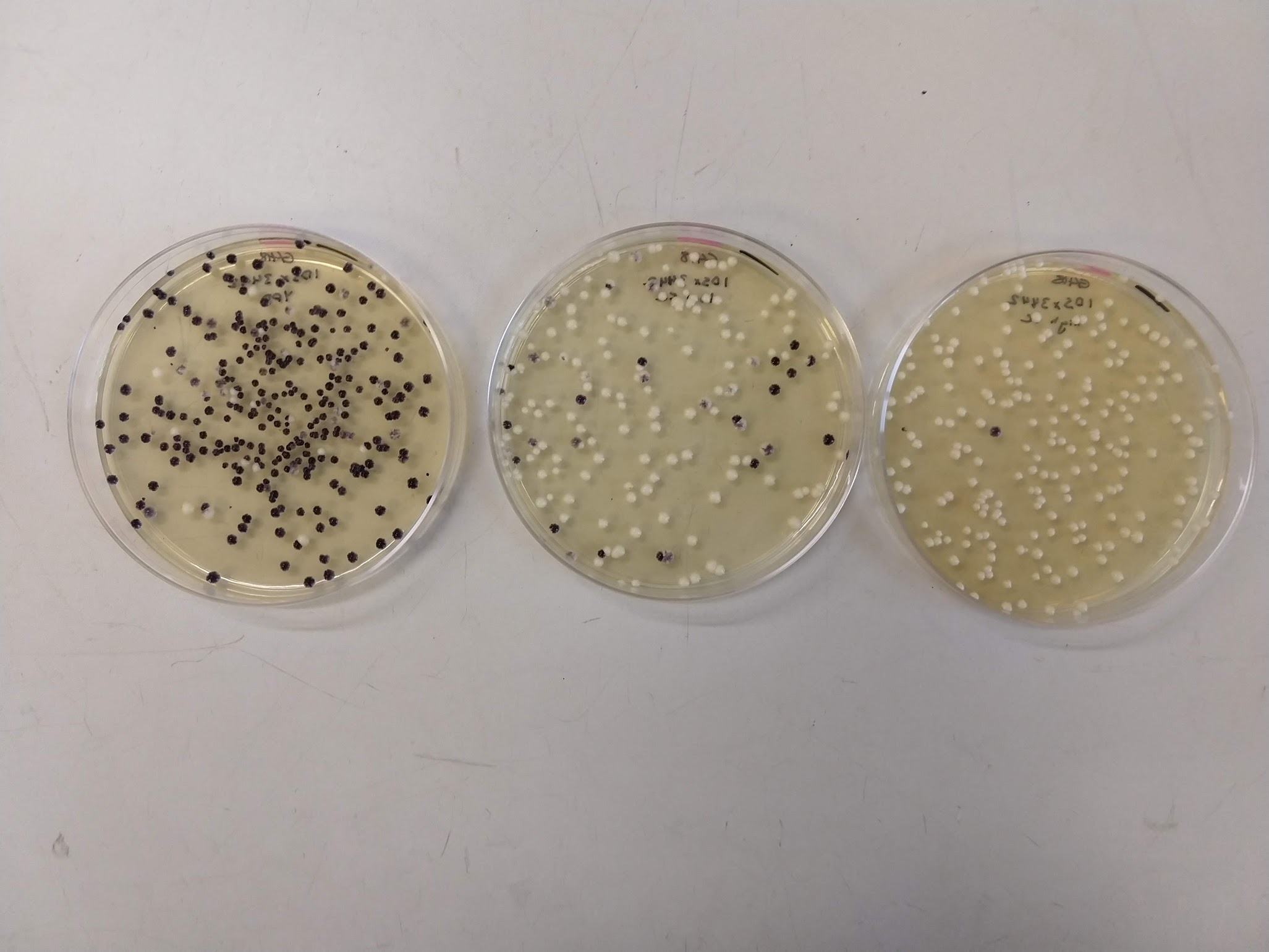


**Figure 2:** **Example of competition experiment outcome.** A black ancestral strain and a cream evolved strain were co-cultured in YPD + G418 media containing no FungiCure, a low dose, and a high dose for 24 hours. A 1:10,000 dilution of each culture was plated onto YPD + G418 agar media. Left plate: no FungiCure; middle plate: low dose (2.25µM); right plate: high dose (9µM).

**GLOSSARY**

- CFU: Colony Forming Unit; a cell that is capable of growing into a colony of cells when transferred from liquid to solid medium. CFUs are commonly used as a proxy for the number of viable cells in a liquid culture.
- Clotrimazole: An azole antifungal. Inhibits synthesis of ergosterol, a key membrane component and the fungal equivalent of cholesterol. Clotrimazole is the active ingredient in the FungiCure spray used in this experiment.
- Fitness: A measure of an individual’s reproductive success. Note that in this experiment you will not be measuring fitness directly but will instead observe the outcome of competition between individuals with differing fitness in a given environment. Success in this competition is determined by the relative fitness between these individuals.
- G418: Geneticin; an antibiotic commonly used in laboratory experiments. Yeast utilized in this protocol are resistant to G418 due to a plasmid they carry, which also gives them their distinctive color thanks to additional genes on the plasmid that encode pigment production pathways. G418 is necessary for maintenance of the plasmid and additionally helps to prevent contamination.
- Selection pressure: An environmental condition that favors some genotypes in a population over others.
- YPD: A standard rich yeast medium named for its three ingredients: Yeast extract, Peptone, and Dextrose. Also referred to as YEPD.

**MATERIALS AND EQUIPMENT**

Yeast strains

- Evolved and ancestral *S. cerevisiae* strains (from Module 1) carrying different pigment expression plasmids

Equipment

- Pipettes: volume needs will vary based on implementation. You will need a P2-20ul, a P20-200ul, and a P200-1000ul or equivalent, as well as a 5ml serological pipette.
- Culture tubes
- Glass beads or plate spreader

Consumables

- YPD + G418 liquid media (at least 30ml experiment)
- YPD + G418 agar plates (at least 4 per experiment)
- Eppendorf tubes
- Sterile swabs, sterile inoculating loops, or sterile inoculating sticks

Chemicals

- FungiCure spray with active ingredient clotrimazole

*Optional*

- *30^o^C incubator*
- *Test tube roller drum or shaking platform*
- *Vortex machine*

**BEFORE THE LAB**

1. Plan out how the timing of activities will fit with your class schedule. Yeast grow most robustly at 30^o^C. They can be grown at room temperature as well but will grow more slowly. *We’ve included estimates for the time it’ll take for your students’ yeast to grow where applicable in italics*.
2. Make FungiCure media. Each competition performed will require 5ml each of a low and a high dose of FungiCure media. In our hands, the following concentrations work well:

- Low dose: 1:12,800x, 2.25µM (3.88ul of fungicure in 50ml of YPD + G418) inhibits growth the ancestral strains and was the starting concentration for the evolutions.
- High dose: 1:3200x, 9µM (15.5ul of fungicure in 50ml of YPD + G418) prevents growth of the ancestral strains but not the evolved strains.
- Very high dose: 1:800x, 36µM (62.1ul in 50ml of YPD + G418) prevents growth of the ancestral strains and several evolved strains, but some evolved strains will grow.

1. Streak evolved and ancestral strains onto YPD + G418 agar media at least 2 days before the intended start of the lab.

**PROTOCOL**

**Day 1:** Inoculate evolved and ancestral strains of yeast into separate tubes of medium.

1. Fill two test tubes with 5ml each of liquid YPD + G418. Label one “evolved” and the other “ancestor”.
2. Use a sterile swab, inoculating loop, or inoculating stick to pick a colony of either evolved or ancestral yeast and inoculate it into its respective test tube.
3. Allow the yeast in these tubes to grow until you can no longer see through the liquid media. *When growing at 30^o^C in a roller drum or shaking platform, this will take 1-2 days. When growing on a bench top at room temperature without shaking or rolling it will take 2-3 days. Yeast can be left longer than these amounts of time (up to a week) without worry*.

**Day 2:** Mix evolved and ancestral strains in media with or without FungiCure.

1. Fill one test tube each with 5ml of the following three media: YPD + G418; YPD + G418 + low dose of FungiCure; YPD + G418 + high dose of FungiCure; YPD + G418 + very high dose of FungiCure. Label these tubes with the media type used.
2. Examine the cultures you inoculated on Day 1. If yeast have settled at the bottom of the tube (pelleted), gently shake the tube until they are completely resuspended. The liquid sample of yeast (culture) should be dense enough that you cannot see through it, and the two cultures should be comparable in density.
3. Remove 20ul of each culture and mix them together in a single eppendorf tube. Make sure to mix well by vortexing or pipetting up and down several times.
4. Add 5ul of mixed culture to each of the three test tubes you inoculated. *Incubate these as on Day 1.*
5. Prepare a 1:10,000 dilution of the remaining mixed yeast culture via serial dilution. Fill two eppendorf tubes with 990ul water and label them “dilution 1” and “dilution 2”. In the tube labeled “dilution 1”, add 10ul of mixed culture and vortex for 5 seconds or invert 10 times to mix. Transfer 10ul from “dilution 1” to “dilution 2” and vortex for 5 seconds to mix.
6. Use a sterile plate spreader or glass beads to spread 150ul from “dilution 2” onto a YPD + G418 agar plate. *Allow this plate to grow until colonies have formed and their colors can be clearly distinguished (2-3 days at 30^o^C; 3-4 days at room temperature). Plates can be left at 30^o^C for 5 days or room temperature for a week without worry.*

**Day 3:** Plate mixed cultures onto YPD + G418 agar media.

1. Examine the cultures you inoculated on Day 2. If yeast have settled at the bottom of the tube (pelleted), gently shake the tube until they are completely resuspended. The liquid sample of yeast (culture) should be dense enough that you cannot see through it, though the culture grown in the highest dose of FungiCure may appear less dense than the others.
2. Prepare a 1:10,000 dilution of each mixed yeast culture via serial dilution. For instance, for the YPD + G418 media condition, fill two eppendorf tubes with 990ul water and label them “YPD + G418 dilution 1” and “YPD + G418 dilution 2”. In the tube labeled “YPD + G418 dilution 1”, add 10ul of mixed culture and vortex for 5 seconds or invert 10 times to mix. Transfer 10ul from “YPD + G418 dilution 1” to “YPD + G418 dilution 2” and vortex for 5 seconds to mix.
3. Use a sterile plate spreader or glass beads to spread 150ul from “YPD + G418 dilution 2” onto a YPD + G418 agar plate. *Allow this plate to grow until colonies have formed and their colors can be clearly distinguished (2-3 days at 30^o^C; 3-4 days at room temperature).*

**Day 4:** Calculate ratio of colors among CFUs as a proxy for relative fitness.

1. Count the number of colonies on each plate onto which you spread yeast culture.
2. Consider the following questions.

**QUESTIONS**

- - - 1. Do you see an equivalent number of colonies of each color on the plate you made on Day 2?
      2. Does the color ratio differ across the three media conditions you plated from on Day 3?
      3. Which strain produced the most colonies after competition in YPD + G418?
      4. Which strain produced the most colonies after competition in YPD + G418 + a high dose of FungiCure?

**EXTENSION QUESTIONS**

1. How do your results compare to those of other groups?
2. Do yeast with a high fitness in one environment (presence of FungiCure) always have a high fitness in other environments (absence of FungiCure)?
3. Do all yeast adapted to the same environment (evolved in the same dose of FungiCure) have the same fitness in that environment?

**Azole Resistance Module 5**
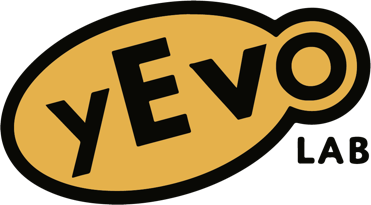


**Metabolic Tradeoffs**

**GOALS**

1. Observe metabolic phenotypes by growing yeast on different media.
2. Understand that increased fitness in one environment may come at the expense of decreased fitness in a different environmental condition.
3. Measure the frequency of metabolic tradeoffs in azole-resistant yeast.

**OVERVIEW**

Yeast, like all living organisms, need a source of carbon in their diet. Yeast are most commonly fed a sugar called dextrose, which is the D in YPD growth medium. The evolution experiments that produced the azole-resistant strains you will work with in this module used YPD medium. Yeast can process dextrose through cellular respiration and through fermentation. Some mutations can prevent yeast from undergoing cellular respiration, such as loss of their mitochondrial genome. These mutations prevent growth on non-fermentable carbon sources (e.g. ethanol or glycerol) and lead to slower growth on fermentable carbon sources like dextrose. Because of this slower growth phenotype, respiratory-deficient strains produce smaller colonies and are referred to as “petite mutants”. It may seem strange that an evolution experiment would produce yeast that grow more slowly. Shouldn’t all mutations that increase fitness lead to faster growth? This paradox is an example of an evolutionary trade-off.

Many of the yeast we’ve isolated from your experiments are petite due to a loss of mitochondrial DNA. Previous work from other labs has demonstrated that petite mutants tend to have a higher resistance to azole drugs, such as the active ingredient in FungiCure (clotrimazole). In this lab, you will estimate the frequency of petite mutations in your experiments. To do this, you will isolate individual cells from your culture by plating them at low density on YPD, so that each cell can grow up and form a genetically-identical colony. You will then transfer these to a YPG plate (glycerol instead of dextrose) and see which ones are capable of utilizing this carbon source.

**GLOSSARY**

- Cellular respiration: metabolic process to release energy from carbon compounds that occurs in the mitochondria and requires oxygen
- Clotrimazole: An azole antifungal. Inhibits synthesis of ergosterol, a key membrane component and the fungal equivalent of cholesterol. Clotrimazole is the active ingredient in the FungiCure spray used in this experiment.
- Fermentation: metabolic process to release energy from carbon compounds that does not require oxygen or mitochondria
- Fitness: A measure of an individual’s reproductive success.
- Petite: small colonies produced by yeast strains that are deficient in cellular respiration
- Selection pressure: An environmental condition that favors some genotypes in a population over others.
- YPD: Yeast Extract, Peptone, and Dextrose; a standard rich yeast medium named for its three ingredients. Also referred to as YEPD.
- YPG: Yeast Extract, Peptone, and Glycerol; a rich yeast medium that contains a non-fermentable carbon source (glycerol). Also referred to as YEPG.

**MATERIALS AND EQUIPMENT**

Yeast strains

- Evolved and ancestral *S. cerevisiae* strains (from Module 1)

Equipment

- Sharpies and rulers

Consumables

- YPD agar plates (1 per strain tested)
- YPG agar plates (1 per strain tested)
- Sterile swabs, sterile inoculating loops, or sterile inoculating sticks

*Optional*

- *30^o^C incubator*

**BEFORE THE LAB**

1. Plan out how the timing of activities will fit with your class schedule. Yeast grow most robustly at 30^o^C. They can be grown at room temperature as well but will grow more slowly. *We’ve included estimates for the time it’ll take for your students’ yeast to grow where applicable in italics*.
2. Streak evolved and ancestral strains onto YPD + G418 agar media at least 2 days before the intended start of the lab.

**PROTOCOL**

**Day 1:** Grow evolved and ancestral strains of yeast on permissive YPD media.

1. Streak yeast from one of your ancestral and one or more of your evolved populations onto a YPD plate.
2. Let them grow until you can clearly distinguish colonies on the plate. *When growing at 30^o^C, this will take 1-2 days. When growing on a bench top at room temperature it will take 2-3 days. Yeast can be left longer than these amounts of time (up to a week) without worry*.

**Day 2:** Grow evolved and ancestral strains of yeast on selective YPG media.

1. Use a sharpie and a ruler to draw a grid on the back of a YPG (glycerol) plate. The grid should have at least 10 evenly-sized boxes. See **Figure 1A** below for an example with 12 evenly-sized boxes.
2. Use a sterile utensil to pick 10 colonies and “patch” them onto your YPG plate by spreading them evenly within the outline of one of the boxes you drew. You don’t need to cover the entire box—in fact it’s best to leave a little space at the edges so cells from one box don’t intrude on a neighboring box. See **Figure 1B** below for an example.

*To get a good estimate of frequency, it’s important to come up with a scheme that reduces experimental bias. This could be “I chose the last 10 colonies from my streak”, or “half my colonies were large and half were small, so I picked 5 large and 5 small colonies”.*

1. Record the phenotype of each patched colony (was it larger or smaller than other colonies on the plate?).
2. Allow yeast to grow on YPG plate for 2-7 days.


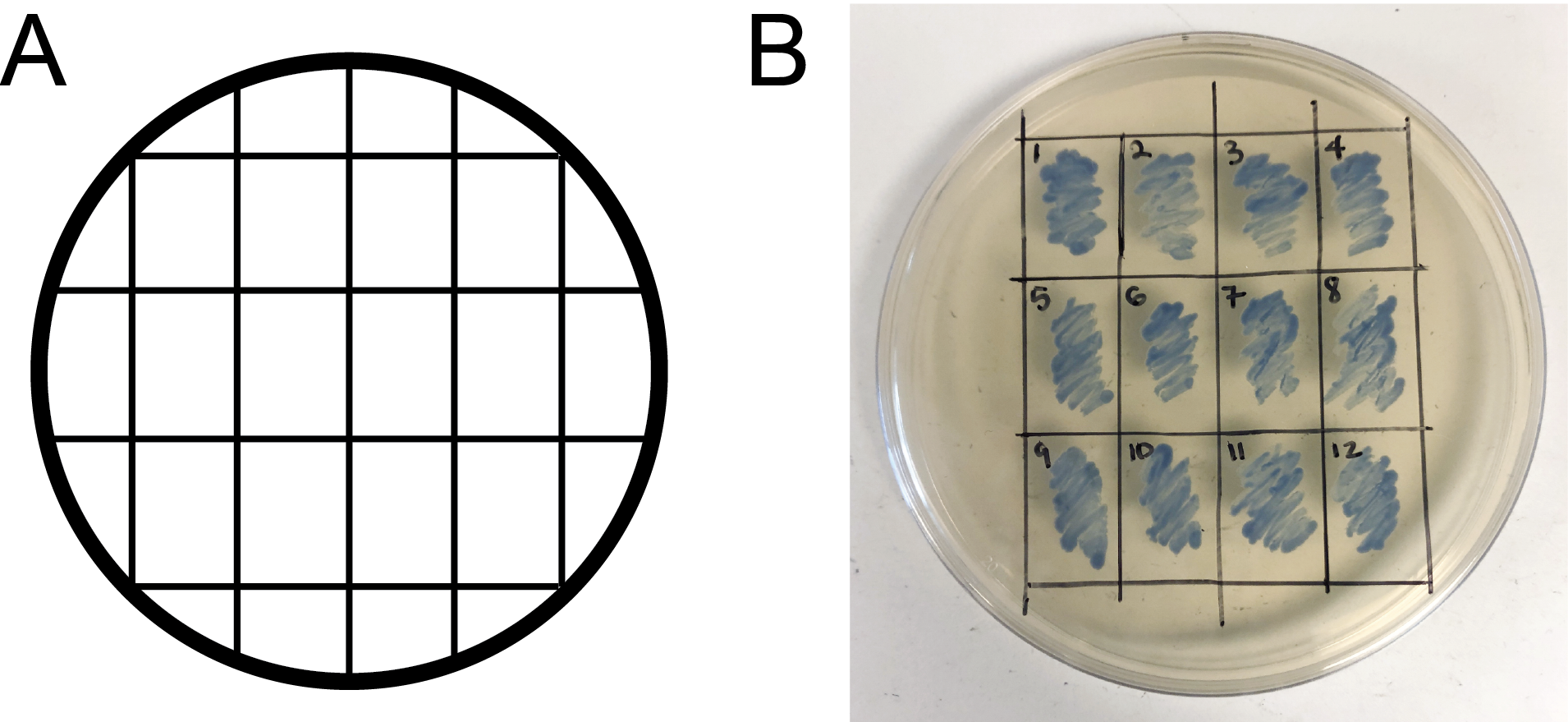


**Figure 1: Patching colonies.** (A) Example of a 12-box grid on a plate. (B) Colonies of a blue yeast strain patched into 12 boxes on a YPD plate. Note that space is left around each patch.

**Day 3:** Record phenotypes from growth on YPG.

1. After you can clearly see colonies, answer the following questions.

**QUESTIONS**

- - - 1. What fraction of patched colonies grew on YPG?
      2. What fraction of patched colonies were petite?
      3. Do phenotypes on YPD plates correlate with any phenotypes you observed on YPG plates?

**EXTENSION QUESTIONS**

1. How would your results differ if you had used a YPG instead of a YPD plate on Day 1?
2. Do you see variability in petite frequency between your evolved replicates? How about between your replicates and your classmates?
3. Does the type of media in which you evolved your yeast impact the types of azole resistance mutations that arose during your experiment?
4. Why would a mutation that makes yeast grow slowly be helpful in some conditions?
5. Why would losing cellular respiration be helpful in dealing with FungiCure?

**Supplemental Text 7.** Survey and interview questions used.

**SECTION 1: Survey questions year 1 - California**

**POST ONLY**

Please respond honestly and completely to each of the following questions. Even when you are not sure how to respond you should give your best guess at an answer.

**Part 1 of 4:** The first set of questions ask you about the yeast evolution lab.

Please briefly describe at least one thing you liked and one thing you did not like about each of the three segments of the yeast evolution lab, listed below. Note: If you did not participate in the activity for any reason please write NA.

1. Growing yeast in the presence of fungicure.

o Liked: (1) ________________________________________________

o Disliked: (2) ________________________________________________

2. Analyzing sequence data from your evolved strains.

o Liked: (1) ________________________________________________

o Disliked: (2) ________________________________________________

3. Competing the strains you evolved with those of your classmates.

o Liked: (1) ________________________________________________

o Disliked: (2) ________________________________________________

4. Briefly explain why you liked/disliked those particular aspects of the yeast evolution lab.

Please read the statements below and indicate your level of agreement with each one.

Options: Strongly Disagree, Disagree, Uncertain, Agree, Strongly Agree, NA

5a. I would be willing to do this lab activity again because I think it was fun.

5b. I enjoyed participating in this lab.

6a. As a result of participating in this lab I am more interested in becoming a biologist.

6b. As a result of participating in this lab I am more interested in pursuing a career in science, technology, engineering, and/or mathematics.

**Part 2 of 4:** The next set of questions will ask you about antibiotic resistance.

7. How would you explain antibiotic resistance to a fellow student in this class?

Please read the statements below (8a, 9a, 10a, 11a) and indicate your level of agreement with each one, then explain your answer in the space provided.

Options: Strongly Disagree, Disagree, Agree, Strongly Agree

8a. Individual bacteria develop mutations in order to become resistant to an antibiotic and survive.

8b. In the space below, please explain your answer to 8a with as much detail as possible.

9a. Individual bacteria are genetically similar and equally likely to be killed by an antibiotic.

9b. In the space below, please explain your answer to 9a with as much detail as possible.

10a. Bacteria develop resistance to antibiotics because of changes within humans.

10b. In the space below, please explain your answer to 10a with as much detail as possible.

11a. Antibiotic resistance is an example of evolution.

11b. In the space below, please explain your answer to 11a with as much detail as possible.

**Part 3 of 4:** The next set of questions ask you about what a biologist does.

Please read the statements below and indicate your level of agreement with each one.

Options: Strongly Disagree, Disagree, Uncertain, Agree, Strongly Agree

12. If I had the necessary materials, I could conduct a successful biology experiment.

13. I am confident that I can design a valid biology experiment.

14. I think about the biology I experience in everyday life.

15. The study of biology is only useful when it directly benefits human health or wellbeing.

16. Biologists may make different interpretations based on the same observations.

17. Biologists do NOT use their imagination because it can interfere with scientific reasoning.

18. Experiments that are done under lab conditions can provide information that applies to the real world.

19. The more hypotheses an experiment attempts to test, the better.

**Part 4 of 4:** The final questions ask about your demographic characteristics. We also ask you to include your name so your teacher can give you credit for your earlier answers.

20. Your first and last name

21. How old are you?

Dropdown options: 13, 14, 15, 16, 17, 18 , 19, 20

22. Which is your gender identity?

o Male

o Female

o Other ________________________________________________

23. Which best describes your race/ethnicity? Select any that apply.

▢ White

▢ Black or African American

▢ American Indian or Alaska Native

▢ Asian

▢ Native Hawaiian or Pacific Islander

▢ Hispanic, Chicanx, or Latinx

▢ Bi/mulit-racial

▢ Other ________________________________________________

24. What is the primary language you speak at home?

25. What is your teacher's last name?

**SECTION 2: Survey questions year 2 - California**

**PRE & POST (any differences indicated)**

In this survey you will be asked to respond to four short question sets. Please respond honestly and completely to each question. Even when you are not sure how to respond you should give your best guess at an answer.

**Part 1 of 4:** The first set of questions ask you some general questions about your biology background.

1. Please describe what you think a yeast is. Again, it’s ok if you aren’t sure, just give your best guess.

2a. Have you had any previous lab experience? **[PRE]**

o YES

o NO

Display This Question:

If 2a. Have you had any previous lab experience? = YES

2b. Please briefly describe your previous lab experience.

3a. Have you taken a biology course before this one? **[PRE]**

o YES

o NO

Display This Question: **[PRE]**

If 3a. Have you taken a biology course before this one? = YES

3b. Please give the course name(s) and grade level(s). **[PRE]**

Please read the statements below and indicate your level of agreement with each one.

Options: Strongly Disagree, Disagree, Uncertain, Agree, Strongly Agree

4. I am interested in becoming a biologist.

5. I am interested in pursuing a career in science, technology, engineering, and/or mathematics.

**Part 2 of 4:** The next set of questions will ask you about microorganisms and antibiotic resistance.

6. How would you explain antibiotic resistance to a fellow student in this class?

Please read the statements below and indicate your level of agreement with each one (7a, 8a, 9a, 10a, 11a), then explain your answer in the space provided.

Options: Strongly Disagree, Disagree, Agree, Strongly Agree

7a. Individual microorganisms develop mutations in order to become resistant to an antibiotic and survive.

7b. In the space below, please explain your answer to 7a with as much detail as possible.

8a. Individual microorganisms are genetically similar and equally likely to be killed by an antibiotic.

8b. In the space below, please explain your answer to 8a with as much detail as possible.

9a. Microorganisms develop resistance to antibiotics because of changes within humans.

9b. In the space below, please explain your answer to 9a with as much detail as possible.

10a. Antibiotic resistance is an example of evolution.

10b. In the space below, please explain your answer to 10a with as much detail as possible.

11a. Scientific findings in microorganisms may be applicable to our understanding of other organisms.

11b. In the space below, please explain your answer to 11a with as much detail as possible.

**Part 3 of 4:** The next set of questions ask you about what a biologist does.

Please read the statements below and indicate your level of agreement with each one.

12. If I had the necessary materials, I could conduct a successful biology experiment.

13. I am confident that I can design a valid biology experiment.

14. I think about the biology I experience in everyday life.

15. The study of biology is only useful when it directly benefits human health or wellbeing.

16. Biologists may make different interpretations based on the same observations.

17. Biologists do NOT use their imagination because it can interfere with scientific reasoning.

18. Experiments that are done under lab conditions can provide information that applies to the real world.

19. The more hypotheses an experiment attempts to test, the better.

**Part 4 of 4:** The final questions ask about your demographic characteristics. We also ask you to include your name so your teacher can give you credit for your earlier answers.

20. Your first and last name

21. How old are you?

Dropdown options: 13, 14, 15, 16, 17, 18 , 19, 20

22. Which is your gender identity?

o Male

o Female

o Other ________________________________________________

23. Which best describes your race/ethnicity? Select any that apply.

▢ White

▢ Black or African American

▢ American Indian or Alaska Native

▢ Asian

▢ Native Hawaiian or Pacific Islander

▢ Hispanic, Chicanx, or Latinx

▢ Bi/mulit-racial

▢ Other ________________________________________________

24. What is the primary language you speak at home?

25. What is your teacher's last name?

**SECTION 3: Survey questions year 2 - Idaho**

**PRE**

**Biology concepts**

Answer the following questions to the best of your ability. Where space is provided, respond in 1-3 sentences.

1. What is a gene?

2. What is a mutation?

3. How would you describe evolution?

4.What role do mutations play in evolution?

5. How would you explain antibiotic resistance to a fellow student in this class?

6a. Individual microbes develop mutations in order to become resistant to an antibiotic and survive.

Options: Strongly disagree, disagree, agree, strongly agree

6b. In the space below, please explain your answer to the question above with as much detail as possible.

7a. Antibiotic resistance is an example of evolution.

Options: Strongly disagree, disagree, agree, strongly agree

7b. In the space below, please explain your answer to the question above with as much detail as possible.

**Personal views**

Respond to each with the following scale: Strongly disagree, disagree, uncertain, agree, and strongly agree

8. I am interested in a career in biology.

9. I am interested in a career in science, technology, engineering, and/or mathematics.

10. I am confident that I can design a valid biology experiment.

11. I think about the biology I experience in everyday life.

12. The study of biology is only useful when it directly benefits human health or wellbeing.

13. Biologists may make different interpretations based on the same observations.

14. Experiments that are done under lab conditions can provide information that applies to the real world.

15. What is your first and last name?

16. What is your age?

16. Which is your gender identity?

o Male

o Female

o Other

o Prefer not to say

23. Which best describes your race/ethnicity? Select any that apply.

▢ White

▢ Hispanic, Chicanx, or Latinx

▢ Black or African American

▢ American Indian or Alaska Native

▢ Native Hawaiian or Pacific Islander

▢ Asian

▢ Bi/mulit-racial

▢ Other

24. What is the primary language you speak at home?

25. What is your teacher's name?

**POST**

Please answer all questions in your own words. Do not use using outside sources (such as Google) to answer these questions.

The questions are intended to assess your understanding and opinions of scientific concepts related to the yeast evolution lab activity. There is no right or wrong answer, you are not being tested!

**Biology concepts**

Answer the following questions to the best of your ability. Where space is provided, respond in 1-3 sentences.

1. What is a gene?

2. What is a mutation?

3. How would you describe evolution?

4.What role do mutations play in evolution?

5. How would you explain antibiotic resistance to a fellow student in this class?

6a. Individual microbes develop mutations in order to become resistant to an antibiotic and survive.

Options: Strongly disagree, disagree, agree, strongly agree

6b. In the space below, please explain your answer to the question above with as much detail as possible.

**Personal views**

Respond to each with the following scale: Strongly disagree, disagree, uncertain, agree, and strongly agree

8. I am interested in a career in biology.

9. I am interested in a career in science, technology, engineering, and/or mathematics.

10. I am confident that I can design a valid biology experiment.

11. I think about the biology I experience in everyday life.

12. The study of biology is only useful when it directly benefits human health or wellbeing.

13. Biologists may make different interpretations based on the same observations.

14. Experiments that are done under lab conditions can provide information that applies to the real world.

**Lab activity experience**

The following questions are about your experience with the yeast evolution lab.

15. Briefly explain something (if anything) you liked about growing yeast in the presence of FungiCure.

16. Briefly explain something (if anything) you disliked about growing yeast in the presence of FungiCure.

[Options: Strongly Disagree, Disagree, Uncertain, Agree, Strongly Agree]

17. I would be willing to do this lab activity again because I think it was fun.

18. I enjoyed participating in this lab .

19. As a result of participating in this lab I am more interested in becoming a biologist.

20. As a result of participating in this lab I am more interested in pursuing a career in science, technology, engineering, and/or mathematics.

**Demographic questions**

21. What is your first and last name?

22. What is your age?

23. Which is your gender identity?

o Male

o Female

o Other

o Prefer not to say

24. Which best describes your race/ethnicity? Select any that apply.

▢ White

▢ Hispanic, Chicanx, or Latinx

▢ Black or African American

▢ American Indian or Alaska Native

▢ Native Hawaiian or Pacific Islander

▢ Asian

▢ Bi/mulit-racial

▢ Other

25. What is the primary language you speak at home?

26. What is your teacher's name?

**SECTION 4: Interview protocol - students**

Evolution learning

1. How would you explain the yeast evolution lab you completed to someone in your family who isn’t a scientist or isn’t familiar with the lab?
2. Thinking back the DNA sequencing part of the lab, how would you describe one of the mutations you found in your evolved yeast?
3. What did you like the most about the lab activity?
4. What did you dislike or like the least about the lab activity?
5. Describe what you think a yeast is (or how would you describe yeast to someone else)
6. Describe what you think evolution is (or how would you describe evolution to someone else)
7. Unpacking terms: adaptation, natural selection
8. Does an organism evolve? Does a species evolve?
9. Biology/STEM career:
   1. For those who said agree/strongly agree to interested in biology/STEM career: Why are you interested in that career? Was there anything you learned as part of the lab activity that interested you in that career?
   2. For those who said disagree/strongly disagree: What career are you interested in? Was there anything you learned as part of the lab activity that you think might help you in that career?
10. What are you future plans? (College, major)
11. For any who ‘agreed’ that biologists do not use their imagination: Tell me a bit more about that answer.

**SECTION 5: Follow up questions (written) - students**

1. What do you remember about the yeast lab?
2. Was it a valuable exercise? Explain why or why not.
3. How would you improve the lab to make it better or more relevant?
4. What career path(s) are you considering pursuing?
5. What grade are you in?

**SECTION 6: Interview protocol - teachers**

**Part 1: Please describe your classroom / school.**

1. What course? What grade level?

2. How many students per class?

3. What biology preparation have students had previously (before your course in which you used yEvo)?

4. Any access or information about students / demographics/ grades/ scores, like SAT etc.? Career paths taken after high school?

**Part 2: How did you get involved in yEvo?**

5. What were you excited to teach about as part of the project?

6. What preparations did you undertake prior to beginning?

**Part 3: How did you integrate yEvo into your course / curriculum?**

7. Walk through the structure / order of yEvo activities you used.

8. What assignments, wrap-up discussions, etc. were assigned in conjunction with those activities? Would you be willing to provide examples of these?

9. How many classroom days / hours / weeks did students participate in yEvo?

10. How did yEvo intersect with your existing course and state/district standards?

11. What did you replace, if anything, to fit it in?

**4. How did it go?**

12. What were students excited about? What went well in the classroom?

13. What student conversations or comments made stood out to you?

14. What was confusing or difficult for students to do or understand?

15. Were your expectations met about your involvement?

16. Do you have any recommendations for how we can improve this activity going forward?

1*7*. What do you plan to do in the future with regard to yEvo?

**Supplemental Text 8.** Extended evaluation.

**Methods**

*Survey development.* We developed an online survey using published questions (Jeffery et al. 2016; Richard et al. 2017) and novel yEvo-specific questions related to the modules (**Supplemental Text 7**). Our goals were to evaluate (1) how students conceptualized topics introduced through yEvo, (2) which aspects of yEvo students liked and disliked, (3) how yEvo impacted students' confidence in their ability to perform scientific investigations, and (4) changes in students' interest in STEM and biology-related careers. Interview questions were developed to follow up with student responses to survey questions via remote interviews (on Zoom; **Supplemental Text 7**). Prior to any module activities, parents/guardians were sent a handout with information about the study and a form to return if they did not consent to share their child's data for the study (passive consent). Students also assented to share their data through either a hardcopy or online form. Teachers responded to interview questions to clarify their classroom activities and capture feedback for future iterative improvements via remote interviews (**Supplemental Text 7**). All research methods were submitted and determined to qualify for exempt status by the University of Washington (IRB #00003148).

*Survey data collection.* The modules and surveys were implemented across high school classrooms from 2017 through 2020 (**Supplemental Figure 1**). In year one, we only administered a post-lab survey in California to test the survey questions on this new population and receive initial student feedback to make adjustments to modules for year two. In year two we administered both pre- and post-lab surveys at both schools and conducted semi-structured interviews with a subset of California students from both school years. Because of differences in module usage resulting from school closure during the COVID-19 pandemic, we report our results for the two teachers in Idaho separately unless noted.

We also collected the AP Biology test scores from 2014-2019 classes at the California school. We calculated a weighted score for the class using the formula 1/2(mean multiple-choice score)+1/2(mean free response question score). We additionally calculated a global average for all who took this exam. The values shown are the differences between those scores.

*Analysis of student evaluations.* Quantitative survey responses were analyzed by comparing the teacher group averages and individual student changes in the pre- and post-survey using t-tests. To directly compare pre- and post-changes, we used only paired pre-post responses for some analyses. We used averages of all collected responses for other analyses, even if a student did not complete both surveys. Short-answer survey questions were coded for response themes using qualitative content analysis, primarily a summative approach (Hsieh and Shannon 2005). We developed a coding scheme to pull key terms or phrases from open-ended responses to questions 1-4 and 6 (**Table 2**) by reviewing learning objectives based on NGSS, drafting an ideal correct response, and then reading a subset of student responses for emergent themes. Thus, we also included three codes intended to capture responses indicative of an incomplete understanding or misconception ('make' in Q1; 'vague adapt' and 'naive' in Q3). We include a summary of code names and descriptions (**Supplemental** **Tables 3-7**). Student responses were coded in a binary system, with '0' meaning the student did not include the key term or concept in their response and '1' meaning the student did include the key term or concept in their explanation. Student responses could include zero or multiple codes for a single question. In contrast to the other questions, student responses to question 5 (**Table 2**) were evaluated on a 0-3 point rubric for correctness, summarized in **Supplemental Table 2**. All codes and the scoring rubric were iteratively developed among three co-authors (multiple rounds of inter-rater coding comparisons) and coded blind with regard to student classroom and pre-post status. For analysis, most Likert-response questions were converted to a numerical scale where Strongly Disagree = 1, Disagree = 2, Neutral = 3, Agree = 4, and Strongly Agree = 5.

**Results**

*Patterns in term usage pre-post.* Our survey included four open-ended questions (Q1-Q4) about genetics and evolution concepts related to yeast evolution. Across all student responses, we noticed an increase in the use of several themes related to more precise terminology from the pre- to post-survey (**Supplemental Tables 8-12; Supplemental Figure 3**). For example, on Q1 (*What is a gene?*), we saw a significant increase in the term "code." Interestingly, more students in the post-survey referred to genes as having a "code" rather than using more vague terminology that we grouped under the theme "make" (**Supplemental Table 8; Supplemental Figure 3**), whereas, in the pre-lab survey, students were more likely to use "make" than "code." This result may suggest a more precise understanding of gene and DNA structure and function. On Q3 (*How would you describe evolution?*), students completing the post-survey were more likely (when compared to their pre-survey) to reference "slow" and "mutation". They were less likely to include common misconceptions, which we captured under "naive" (**Supplemental Table 10; Methods**). This result supports a more precise understanding of the concept. Additionally, we measured a decrease in the use of "adapt" on Q3 and an increase in the usage of “variation” on Q4 (*What is the role of mutations in evolution?*).

*Lasting impact on interests in STEM fields.* Since these activities were designed to be true to scientific research practices, we investigated how participation would impact student conceptions of the process of biology (**Supplemental Figure 4**) and interest in STEM fields (**Supplemental Figure 5**). On pre- and post-lab surveys, we asked students about their interest in biology or STEM careers. Students who completed both surveys showed a small average increase in interest over this time period (**Table 5**), though this difference was not significant. We directly asked students whether participating in this activity increased their interest in a STEM or a Biology career on our post-lab surveys. Of students who responded to both the pre- and post-survey, 15/46 (33%) agreed or strongly agreed with one or both questions (**Table 3**). This encouraging result led us to wonder what students would recall one year later.

*Feedback from students one to two years later.* Thirty-nine students from the 2018-2019 and 2019-2020 school years at the Idaho school were surveyed again in 2021 (**Supplemental Table 13**) to check for retention of knowledge gained from the yEvo experiments. When asked about their memory of the lab, students mentioned: "yeast poison" or "acid" to describe the clotrimazole, showing that the students seemed to retain the information of the general experiment but not the specifics of the lab. The majority of students (27/39) responded that the lab was valuable to their experience. Four students felt that their experience lacked an understanding of how the research stemming from their experiments was being used. Two students mentioned losing interest or motivation to continue the repetitive task of transferring yeast over the course of the lab, especially if they were not pushed to continue discussing it. However, ten students mentioned how it demonstrated applications of evolution or simulated real lab experiences: "It mimicked actual lab work, and we got to make real observations” and “We actually got to observe yeast evolving.”


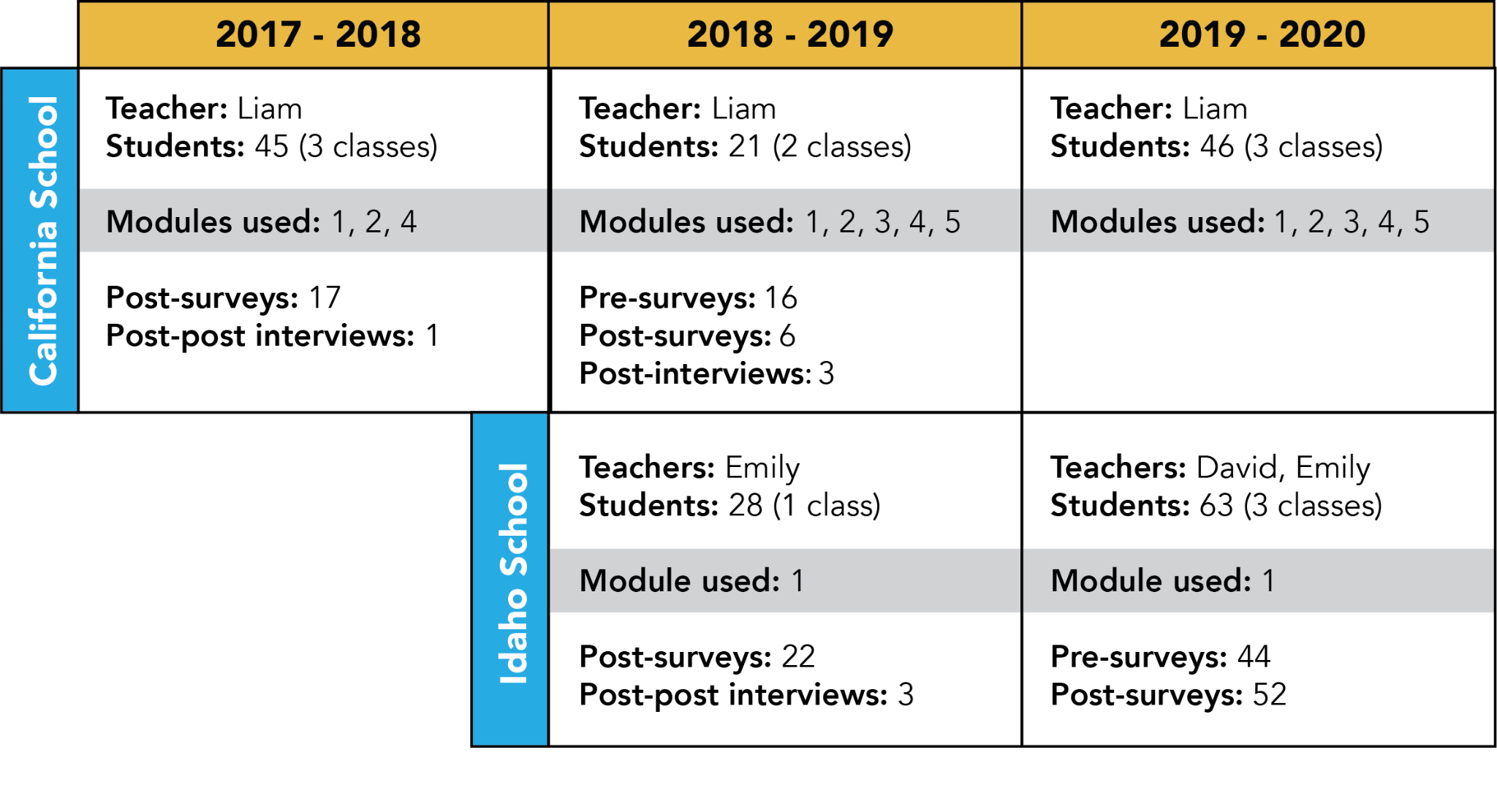


**Supplemental Figure 1.** Timeline of module implementation for each school/teacher, number of students involved, and evaluation collected.


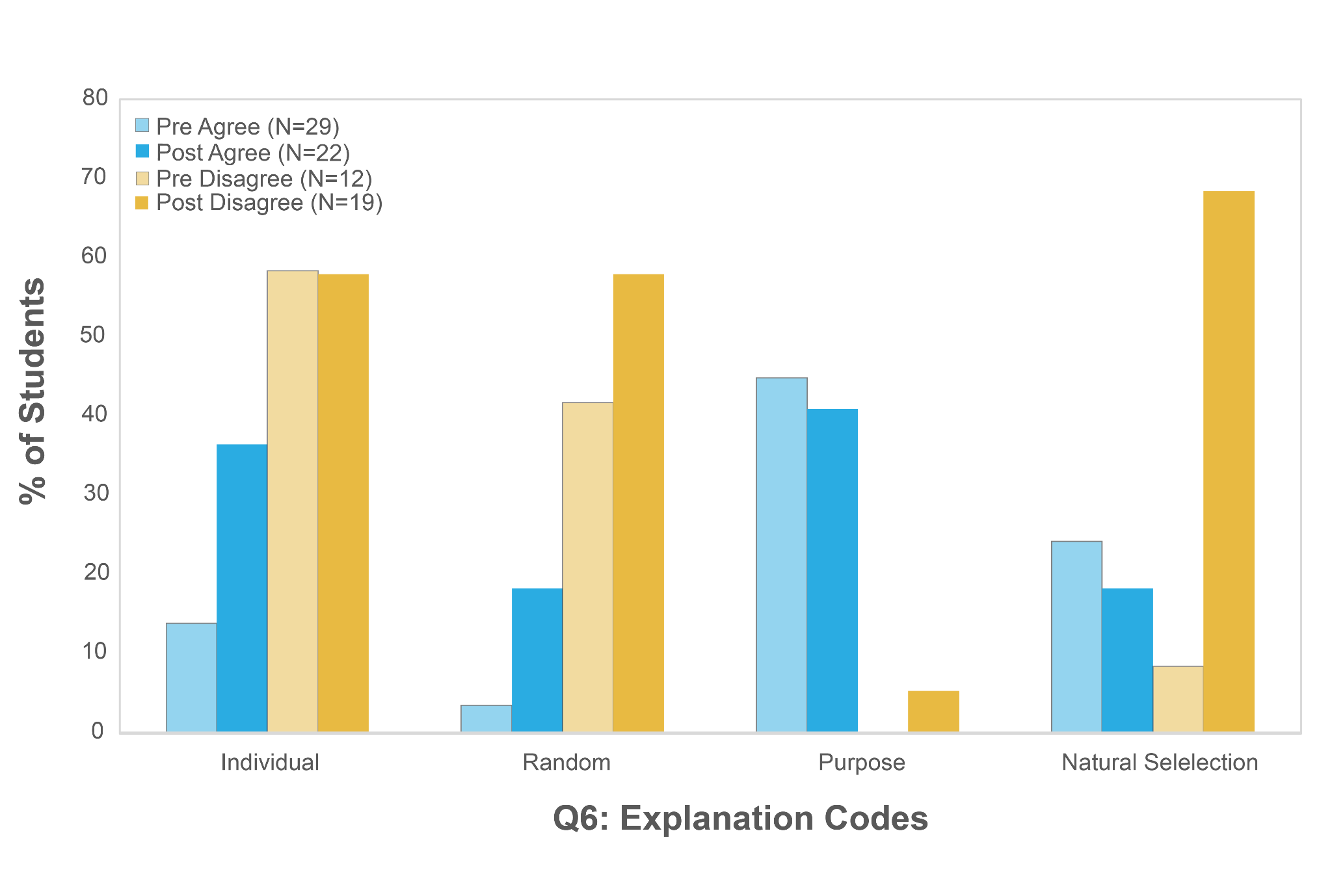


**Supplemental Figure 2.** Percent of student open-ended responses to question 6 (Q6; [*Explain why or why not]* *microbes develop mutations in order to become resistant to an antibiotic and survive*.) coded by four explanations (see **Table S6**) for pre- (lighter shade) and post- (darker shade) surveys. Each student’s response (N = 41) is categorized by their level of agreement to the original statement; agree (blue; either agree or strongly agree) and disagree (yellow; either disagree or strongly disagree).

**
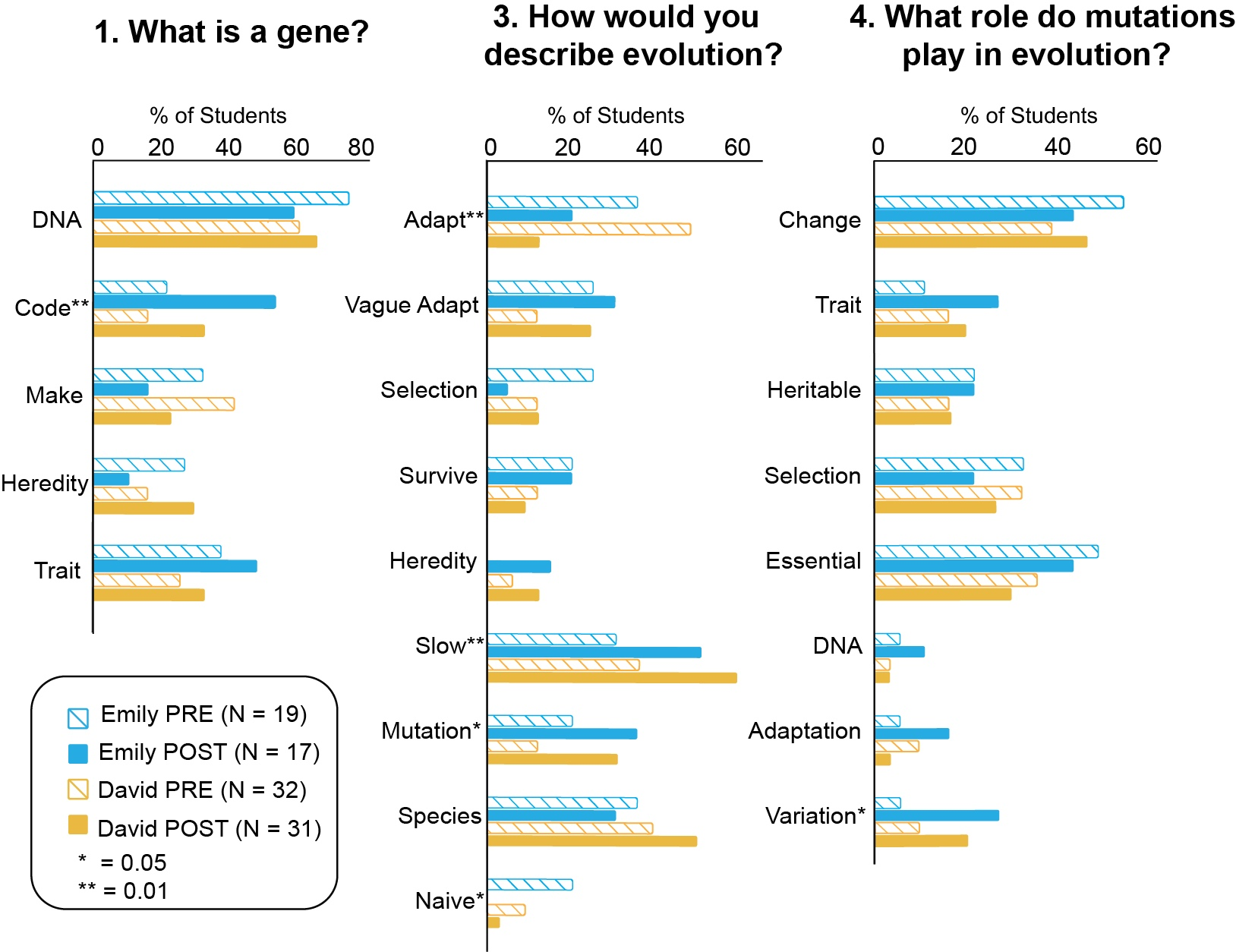
**

**Supplemental Figure 3.** The proportion of the Idaho school students who used key terms and phrases (codes) in their responses to three pre and post-survey questions, grouped by each teacher (Emily, David). Percentages per survey question do not sum to 100% because a single student could have multiple codes per response. Significant differences between pre and post across all students are indicated by * (alpha 0.05 level) and ** (alpha 0.01 level). See p-values in **Supplemental Table 12**.


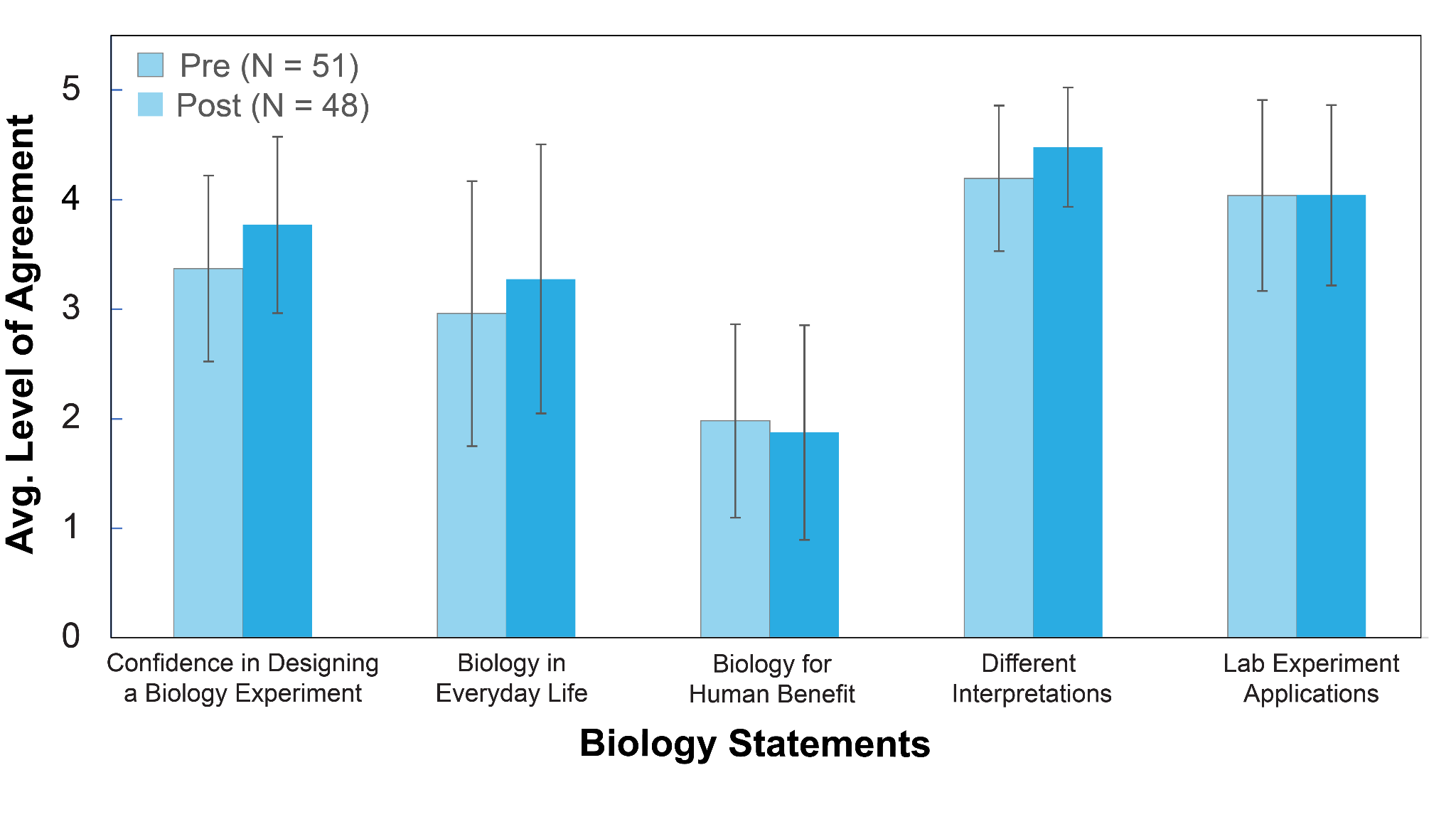


**Supplemental Figure 4.** Average level of agreement (5 = strongly agree) to Likert-response biology statements from students who completed a pre- (N = 51) or a post-lab (N = 48) survey at the Idaho school during the 2019-2020 school year. Standard deviation shown. None of the statements were significantly different from pre to post (t-test, alpha = 0.01).


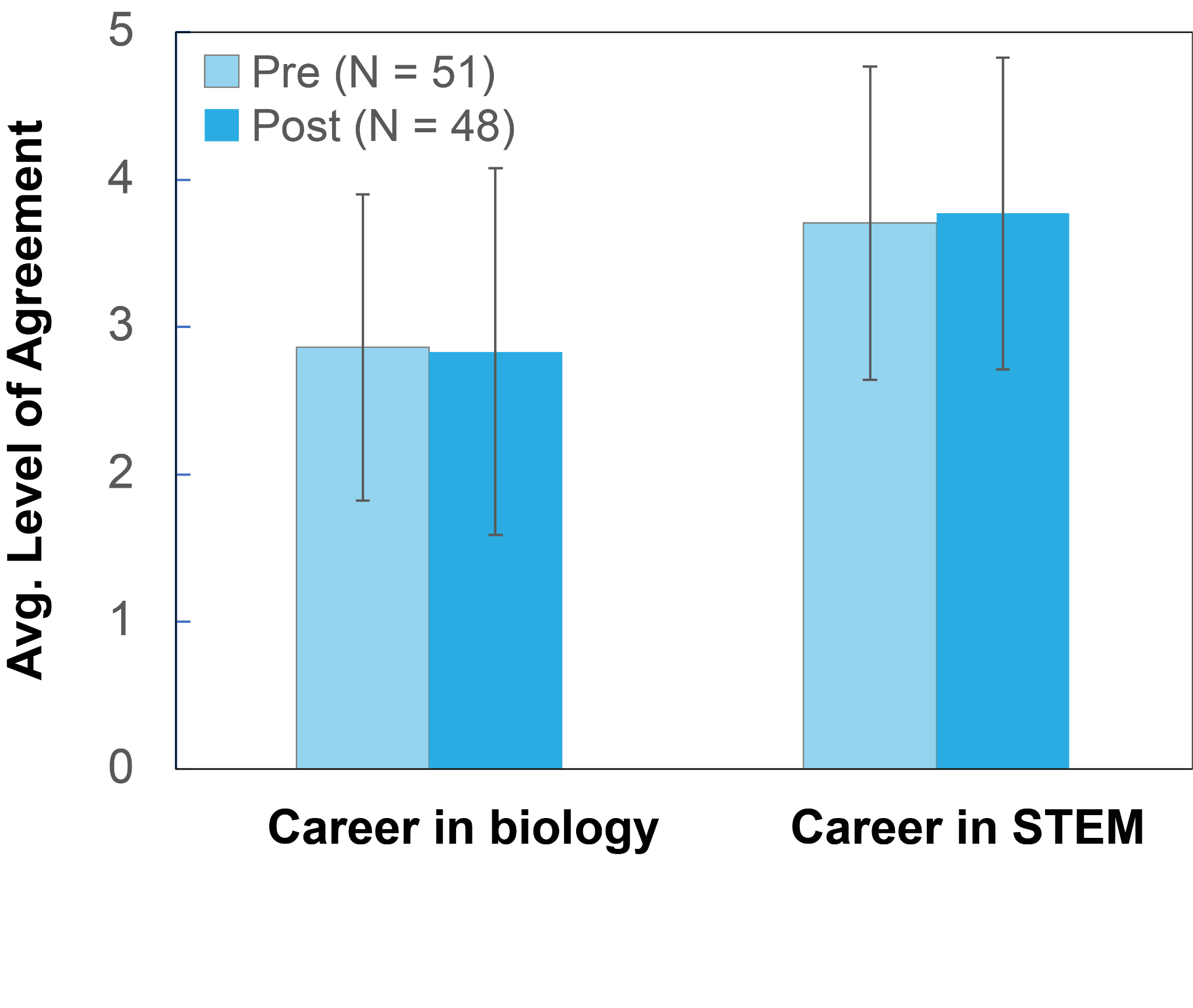


**Supplemental Figure 5.** Average level of agreement (5 = strongly agree) in Idaho school students' interest in either a career in biology or a career in science, technology, engineering or math (STEM) in the pre- (N = 51) or a post-lab (N = 48) survey at during the 2019-2020 school year. Standard deviation shown. Neither of the statements were significantly different from pre to post (t-test, alpha = 0.01).

**Supplemental Table 1.** Self-reported characteristics of participants include the number of unique students with data collected from pre/post surveys for each of the three teachers, gender, race/ethnicity (if multiple race/ethnicity options or multiracial selected then counted under ‘multiple’), the mean age of students (if they responded to both pre- and post-survey we used their age at post), and the primary language spoken at home. Student gender and racial demographics are representative of the school as a whole based on publicly-available demographic records.

| **Teacher (State)** | **No. of Students** | **Gender identity** | **Race/Ethnicity** | **Age (mean)** | **Language(s) spoken at home** |
| --- | --- | --- | --- | --- | --- |
| Emily (ID) | 21 | 11 Female  10 Male | 15 White  4 Multiple  3 Asian  1 Unknown | 15.33 | 19 English  2 Other language |
| David (ID) | 37 | 18 Female  18 Male  1 Other | 31 White  4 Multiple  1 Hispanic | 15.69 | 36 English  1 Multilingual |
| Liam (CA) | 14 | 14 Female | 7 Multiple  5 White  1 Asian | 16.35 | 11 English  2 Multilingual  1 Other language |

| **Score** | **Description** |
| --- | --- |
| 0 | Responded that they don’t know, OR the student’s response is entirely / mostly incorrect. |
| 1 | Echoed the question without giving any additional information. An example of this would simply be restating that the bacteria are resistant to the drugs. |
| 2 | Mostly scientifically accurate, but included some misconceptions or only partially explained antibiotic resistance. Student added some but not all of the information needed to explain the phenomena of antibiotic resistance. |
| 3 | Scientifically accurate and explained the concept of antibiotic resistance without any misconceptions. An ideal response mentioned how the microbes aren’t responding to the drugs using key terms or concepts such as - evolution, selection, mutation, and resistance. Explaining key terms without using them is okay. |

**Supplemental Table 2.** Rubric for scoring question 5: How would you explain antibiotic resistance to a fellow student in this class?

**Supplemental Table 3.** List of code descriptions for question 1: What is a gene?

| **Name of code** | **Description** |
| --- | --- |
| DNA | Described gene as made up of DNA, is DNA, or is a section / sequence of DNA. |
| Code | Mentioned that a gene contains information or codes for / determines the traits of an organism. Other verbs allowed including ‘decides’ / ‘determines’ / ‘controls’ traits.  Did not allow ‘associated’. |
| Heredity | Mentioned genes are the unit of heredity or that they are inherited or come from / are passed on by parents. Did not have to use the term heredity or inherited. |
| Trait | Referenced that a gene is a trait or that genes determine traits. Allowed reference to a trait, characteristic, feature, structure, or appearance of an organism. It was not sufficient to say that a gene determines ‘something’ or that it ‘makes you who you are’. Must be more specific. |
| Make | Used the word ‘make’ or other word / phrase that skipped over the process steps (intermediate steps) between gene and trait. Allowed ‘create’. Did not allow ‘information’, as this falls under “code”. Did not allow that a gene is ‘made’ of DNA or ‘genetic makeup’. [S023, S027]. |

| **Code** | **Description** | **Example student response** |
| --- | --- | --- |
| Inherited | Used term(s) such as ‘inherited’, ‘passed down’, or ‘heritable’ when referring to mutations or traits. Allowed responses that implied heritability. | “A heritable adaption of a species.” [S001] |
| Phenotypic | Mentioned a change in phenotype or trait. Included references to traits being physically expressed in a different way. Must reference a physical change. | “A mutation is something that is like unusual to happen like blue eyes” [S002]  “Something gets screwed up in the DNA and something weird happens like pinkies.” [S015] |
| Genotypic | Mentioned any change in genotype. Allowed differences in DNA base pair or nucleotide sequence or mistake / error in copying or replicating DNA. | “A mutation is when one of the Bases pairs up with the wrong base pair. It causes mutation because the two bases don't fit together.” [S011]  “Something gets screwed up in the DNA and something weird happens like pinkies.” [S015] |
| Variation | Mentioned variation among individuals. Allowed use of the phrase ‘genetic variation’ or description of a mutation as something which creates variation. | “A genetic variation” [S041]  “...resulting in a variant form…” [S052] |
| Evolution | Used the term mutation to describe how evolution occurs or as something which contributes to evolution | “A change in the genes of an organism, causing the organism to function slightly differently. These mutations add up over time to create the evolutionary process.” [S036] |

**Supplemental Table 4.** List of code descriptions and example student responses for question 2: What is a mutation?

| **Code** | **Description** |
| --- | --- |
| Adapt | Mentioned terms ‘adapt’, ‘adaptation’, or ‘adapted’ to explain evolution. |
| Vague adapt | Mentioned adaptation without explicit use of the term ‘adapt’. For instance: ‘evolve to their environment’ or ‘changed to fit the environment’. |
| Selection | Used the term ‘natural selection’. |
| Survive | Mentioned survival of organisms, usually regarding increased survival as a result of a particular trait. Allowed reference to the opposite of survival - death - as a result of species with undesirable characteristics. Did not allow ‘stronger’ traits. |
| Heredity | Explicitly mentioned the terms/phrases ‘inheritance’, ‘heredity’ or ‘passing on’ traits. Allowed reproduction or passing traits to offspring, even if they did not specifically mention genetic factors like mutations. Similarly, allowed loss of traits over time (‘breeding out’) because organisms with those traits die off (do not reproduce). |
| Slow | Mentioned evolution as slow or gradual changes with phrases like ‘over many generations’, ‘long time’, ’long process of time’, ‘many generations’, or ‘change over time’. Did not allow ‘process of change’ or ‘several years of adaptations’. |
| Mutation | Mentioned source of variation from mutations. Allowed ‘Genetic changes’ or ‘changes to DNA’. |
| Species | Mentioned a change in how frequent or common a trait or mutation is in the **species** or **population**, or that change is not occurring at the individual level. Allowed group of animals (more descriptive than ‘animals’ or beings). |
| Naive | Naive explanations mentioned ‘need’ (traits that are ‘needed’ to survive) or ‘intention’ (traits that are ‘chosen’ to help an organism survive). |

**Supplemental Table 5.** List of code descriptions for question 3: How would you describe evolution?

**Supplemental Table 6.** List of code descriptions for question 4: What role do mutations play in evolution?

| **Code** | **Description** |
| --- | --- |
| Change | Mentioned mutations lead to changes in individuals or cause differences in organisms. |
| Trait | Referenced a gene is a trait or that genes determine the code for traits. This particular code is more about the reference to a trait, characteristic, feature, structure, or appearance of an organism. It is not sufficient to say that a gene determines codes for ‘something’ or that it ‘makes you who you are’. Must be more specific. |
| Heritable | Stated that mutations are heritable or can be passed down, so any beneficial or deleterious consequence will apply to offspring as well. |
| Selection | Described how mutations can impact survival or the production of more or less progeny. Did not need to use the term ‘selection.’ Did not allow ‘fit’ without additional clarification of differential survival. |
| Essential for evolution | Described how mutations ‘enable’ evolution. Allowed references that mutations are important but did not require an explanation how. Allowed more general language like ‘help’, ‘allow’, and ‘create’ evolution. |
| DNA | Made a reference to changes in DNA. |
| Adaptation | Used the term ‘adaptation’ or ‘adapt’. |
| Variation | Used the term ‘variation,’ described the change in frequency of a trait within the population, or mentioned differences between individuals / organisms within the population. |

**Supplemental Table 7.** List of code descriptions for question 6: [Explain why or why not] individual microbes develop mutations in order to become resistant to an antibiotic and survive.

| **Code** | **Description** |
| --- | --- |
| Individual vs. Population (distinguish) | Made an explicit distinction between processes happening at the individual and group level (such as colonies, species, organisms, populations), OR stated that individuals can or can’t do something, implying that it must happen at the group level. |
| Random | Mentioned that the mutation is developed by accident or the microbes don’t have control over the development of the mutation. Stated that mutation is **not** **intentional** or a conscious decision. |
| Purposeful | Stated that the mutation is needed (or that yeast ‘have to’ acquire a mutation) to survive or to make a microbe(s) resistant. Implied that the mutation happens for a reason. Allowed responses that do not indicate it’s random (e.g. ‘individual microbes will develop resistance…’). |
| Natural selection | Made a reference to differential survival and reproduction. ‘Pass on’ or ‘around’ the group is allowed. Reference to survival needed to be related to differential survival vs. individual survival. Did not allow ‘evolve’ or ‘adapt’ without additional clarification of how these processes occur. |

**Supplemental Table 8.** List of codes, percent responses per teacher, and example student response for question 1: What is a gene?

| **Code** | **Emily Pre** | **Emily Post** | **David Pre** | **David Post** | **Example student response** |
| --- | --- | --- | --- | --- | --- |
| DNA | 73.7 | 57.9 | 59.4 | 64.5 | "Genes are parts of DNA that create our body composition." [S008] |
| Code | 21.1 | 52.6 | 15.6 | 32.3 | "A component of DNA responsible for determining appearances and bodily structures." [S024] |
| Make | 31.6 | 15.8 | 40.6 | 22.6 | "A characteristic that makes up who and what you are." [S019]  "A strand of DNA that decides someone's physical traits" [S034] |
| Heredity | 26.3 | 10.5 | 15.6 | 29.0 | "A thing that determines your traits passed on from your parents." [S033] |
| Trait | 36.8 | 47.4 | 25.0 | 32.3 | "A gene is a strand of DNA which contains information which decides certain characteristics." [S037]  "Genes are made up of DNA. A gene is a physical unit of traits." [S053] |

**Supplemental Table 9.** List of codes, percent responses per teacher, and example student response for question 2: What is a mutation?

| **Code** | **Emily - Pre** | **Emily - Post** | **David - Pre** | **David - Post** | **Example student response** |
| --- | --- | --- | --- | --- | --- |
| Inherited | 5.3 | 0.0 | 6.3 | 12.9 | "A heritable adaption of a species." [S001] |
| Phenotypic | 15.8 | 21.1 | 9.4 | 3.2 | "A mutation is something that is like unusual to happen like blue eyes" [S002]  "Something gets screwed up in the DNA and something weird happens like pinkies." [S015] |
| Genotypic | 100.0 | 78.9 | 84.4 | 100.0 | "A mutation is when one of the Bases pairs up with the wrong base pair. It causes mutation because the two bases don't fit together." [S011]  "Something gets screwed up in the DNA and something weird happens like pinkies." [S015] |
| Variation | 5.3 | 0.0 | 9.4 | 6.5 | “A genetic variation” [S041]  “...resulting in a variant form…” [S052] |
| Evolution | 10.5 | 0.0 | 0.0 | 0.0 | "A change in the genes of an organism, causing the organism to function slightly differently. These mutations add up over time to create the evolutionary process." [S036] |

**Supplemental Table 10.** List of codes, percent responses per teacher, and example student response for question 3: How would you describe evolution?

| **Term** | **Emily - Pre** | **Emily - Post** | **David - Pre** | **David - Post** | **Example** |
| --- | --- | --- | --- | --- | --- |
| Adapt | 36.8 | 21.1 | 50.0 | 12.9 | "A slow process of adapting traits needed to survive in an environment" [S006] |
| Vague adapt | 26.3 | 31.6 | 12.5 | 25.8 | "Adopting physical changes to help live in changing environment." [S008] |
| Selection | 26.3 | 5.3 | 12.5 | 12.9 | "The process of natural selection using mutations to create a beneficial output." [S028] |
| Survive | 21.1 | 21.1 | 12.5 | 9.7 | "A slow change in a species over time to optimize the ability to survive and reproduce." [S029] |
| Heredity | 0.0 | 15.8 | 6.3 | 12.9 | "Mutations that sometimes stick and get passed on." [S020] |
| Slow | 31.6 | 52.6 | 37.5 | 61.3 | "A species slowly changing generation after generation to adapt to it's environment." [S025] |
| Mutation | 21.1 | 36.8 | 12.5 | 32.3 | "a series of mutations over many generations" [S042] |
| Species | 36.8 | 31.6 | 40.6 | 51.6 | "evolution is the change of a species at a population level over time." [S004] |
| Naive | 21.1 | 0.0 | 9.4 | 3.2 | "a group of animals that are changing themselves according to their weather, geography, etc." [S027] |

**Supplemental Table 11.** List of codes, percent responses per teacher, and example student response for question 4: What role do mutations play in evolution?

| **Term** | **Emily - Pre** | **Emily - Post** | **David - Pre** | **David - Post** | **Example** |
| --- | --- | --- | --- | --- | --- |
| Change | 52.6 | 42.1 | 37.5 | 45.2 | "They control how beings change" [S003] |
| Trait | 10.5 | 26.3 | 15.6 | 19.4 | "They make certain individuals more resistant or not resistant to things." [S009] |
| Heritable | 21.1 | 21.1 | 15.6 | 16.1 | "Mutations can be passed into offspring causing population wide evolution" [S011] |
| Selection | 31.6 | 21.1 | 31.3 | 25.8 | "The mutation either help the organism survive and reproduce more or it kills it off because it's unable to survive." [S007] |
| Essential for evolution | 47.4 | 42.1 | 34.4 | 29.0 | "It is what causes evolution, a species can't evolve without some type of mutation." [S013] |
| DNA | 5.3 | 10.5 | 3.1 | 3.2 | "Mutation's role in evolution is, mutations change the DNA of the animal which changes the animal, and these changes help with evolution." [S050] |
| Adaptation | 5.3 | 15.8 | 9.4 | 3.2 | "Mutations create useful adaptations that help animals in their environment" [S005] |
| Variation | 5.3 | 26.3 | 9.4 | 19.4 | "mutations in a populations can lead to change so if there is a species of beetles living on black gravel it starts out with 75 percent white and 25 percent black beetles the white beetles are more likely to be caught so then the black beetles have more babies and over time the white beetles will die out. "[S004] |

**Supplemental Table 12.** Results of t-tests to test for significant differences between pre and post codes from questions 1, 2, 3, and 4

| **Question** | **Code** | **p-value** |
| --- | --- | --- |
| **Q1** | DNA | 0.9900 |
|  | **Code**** | **0.0090** |
|  | Make | 0.0726 |
|  | Heredity | 0.6915 |
|  | Trait | 0.2927 |
|  | | |
| **Q2** | Inherited | 0.6403 |
|  | Phenotypic | 0.8329 |
|  | Genotypic | 0.2736 |
|  | Variation | 0.4448 |
|  | Evolution | 0.1593 |
|  | | |
| **Q3** | **Adapt**** | **0.0019** |
|  | Vague adapt | 0.1810 |
|  | Selection | 0.3039 |
|  | Survive | 0.8798 |
|  | Heredity | 0.0718 |
|  | **Slow**** | **0.0121** |
|  | **Mutation*** | **0.0253** |
|  | Species | 0.5107 |
|  | **Naive*** | **0.0313** |
|  | | |
| **Q4** | Change | 0.7900 |
|  | Trait | 0.2434 |
|  | Heritable | 0.8884 |
|  | Selection | 0.4858 |
|  | Essential for evolution | 0.6996 |
|  | DNA | 0.6039 |
|  | Adaptation | 0.9297 |
|  | **Variation*** | **0.0399** |
